# Characterising thermal water circulation in fractured bedrock using a multidisciplinary approach: a case study of St. Gorman’s Well, Ireland

**DOI:** 10.1007/s10040-021-02393-1

**Published:** 2021-10-05

**Authors:** Sarah Blake, Tiernan Henry, John Paul Moore, John Murray, Joan Campanyà, Mark R. Muller, Alan G. Jones, Volker Rath, John Walsh

**Affiliations:** 1 Dublin Institute for Advanced Studies (DIAS), Merrion Square, Dublin 2, Ireland; 2 Irish Centre for Research in Applied Geosciences (iCRAG), Dublin 4, Ireland; 3 Geological Survey Ireland, Beggars Bush, Haddington Road, Dublin 2, Ireland; 4 Earth and Ocean Sciences, School of Natural Sciences, National University of Ireland Galway, University Road, Galway, Ireland

**Keywords:** Thermal springs, Karst, Geothermal exploration, Geophysical methods, Ireland, Fractured rocks

## Abstract

A hydrogeological conceptual model of the source, circulation pathways and temporal variation of a low-enthalpy thermal spring in a fractured limestone setting is derived from a multidisciplinary approach. St. Gorman’s Well is a thermal spring in east-central Ireland with a complex and variable temperature profile (maximum of 21.8 °C). Geophysical data from a three-dimensional(3D)audio-magnetotelluric(AMT) survey are combined with time-lapse hydrogeological data and information from a previously published hydrochemical analysis to investigate the operation of this intriguing hydrothermal system. Hydrochemical analysis and time-lapse measurements suggest that the thermal waters flow within the fractured limestones of the Carboniferous Dublin Basin at all times but display variability in discharge and temperature. The 3D electrical resistivity model of the subsurface revealed two prominent structures: (1) a NW-aligned faulted contact between two limestone lithologies; and (2) a dissolutionally enhanced, N-aligned, fault of probable Cenozoic age. The intersection of these two structures, which has allowed for karstification of the limestone bedrock, has created conduits facilitating the operation of relatively deep hydrothermal circulation (likely estimated depths between 240 and 1,000 m) within the limestone succession of the Dublin Basin. The results of this study support a hypothesis that the maximum temperature and simultaneous increased discharge observed at St. Gorman’s Well each winter is the result of rapid infiltration, heating and recirculation of meteoric waters within a structurally controlled hydrothermal circulation system.

**Supplementary Information:**

The online version contains supplementary material available at 10.1007/s10040-021-02393-1.

## Résumé

Un modèle conceptuel hydrogéologique de la source, des voies de circulation et de la variation temporelle d’une source thermale à faible enthalpie dans un contexte de calcaire fracturé est dérivé d’une approche multidisciplinaire. St. Gorman’s Well est une source thermale du centre-est de l’Irlande avec un profil de température complexe et variable (maximum de 21.8 °C). Les données géophysiques d’un levé audio-magnétotellurique (AMT) en trois dimensions (3D) sont combinées avec des données hydrogéologiques à intervalles de temps et des informations provenant d’une analyse hydrochimique publiée précédemment pour étudier le fonctionnement de cet intrigant système hydrothermal. L’analyse hydrochimique et les mesures à différentes périodes suggèrent que les eaux thermales s’écoulent à tout moment dans les calcaires fracturés du bassin carbonifère de Dublin, mais présentent une variabilité de débit et de température. Le modèle de résistivité électrique 3D du sous-sol a révélé deux structures importantes: (1) un contact faillé orienté NW entre deux lithologies calcaires; et (2) une faille alignée au Nord, améliorée par dissolution, d’âge cénozoïque probable. L’intersection de ces deux structures, qui a permis la karstification du socle calcaire, a créé des conduits facilitant le fonctionnement d’une circulation hydrothermale relativement profonde (profondeurs estimées vraisemblablement entre 240 et 1,000 m) au sein de la succession calcaire du bassin de Dublin. Les résultats de cette étude appuient l’hypothèse selon laquelle la température maximale et l’augmentation simultanée du débit observés à St. Gorman’s Well chaque hiver sont le résultat d’une infiltration, d’un réchauffement et d’une recirculation rapides des eaux météoriques dans un système de circulation hydrothermale structurellement contrôlé.

## Resumen

Se deriva un modelo conceptual hidrogeológico de la fuente, las vías de circulación y la variación temporal de un manantial termal de baja entalpía en un entorno de caliza fracturada a partir de un enfoque multidisciplinar. Gorman’s Well es un manantial termal en el centro-este de Irlanda con un perfil de temperatura complejo y variable (máximo de 21.8 °C). Los datos geofísicos de un estudio audio-magnetotelúrico (AMT) tridimensional (3D) se combinan con los datos hidrogeológicos de un lapso de tiempo y la información de un análisis hidroquímico previamente publicado para investigar el funcionamiento de este intrigante sistema hidrotermal. El análisis hidroquímico y las mediciones a intervalos de tiempo sugieren que las aguas termales fluyen dentro de las calizas fracturadas de la cuenca carbonífera de Dublín en todo momento, pero muestran variabilidad en la descarga y la temperatura. El modelo de resistividad eléctrica tridimensional del subsuelo reveló dos estructuras prominentes: (1) un contacto de falla alineado al NW entre dos litologías calcáreas; y (2) una falla de disolución incrementada, alineada al N, de probable edad cenozoica. La intersección de estas dos estructuras, que ha permitido la karstificación del lecho rocoso calcáreo, ha creado conductos que facilitan el funcionamiento de una circulación hidrotermal relativamente profunda (probablemente a profundidades estimadas entre 240 y 1,000 m) dentro de la sucesión calcárea de la cuenca de Dublín. Los resultados de este estudio apoyan la hipótesis de que la temperatura máxima y el aumento simultáneo de la descarga observados en St. Gorman’s Well cada invierno son el resultado de una rápida infiltración, calentamiento y recirculación de aguas meteóricas dentro de un sistema de circulación hidrotermal estructuralmente controlado.

## 摘要

通过多学科方法, 建立了裂缝型石灰岩环境中低焓温泉的来源、循环路径和时间变化的水文地质概念模型。圣戈尔曼井是爱尔兰中东部的温泉, 温度分布复杂多变 (最高21.8 °C)。将三维 (3D) 音频-大地电磁 (AMT) 调查的地球物理数据与延时水文地质数据和先前发布的水化学分析信息相结合, 调查了有趣的热液系统运行情况。水化学分析和延时测量表明, 热水一直在石炭系都柏林盆地的裂缝型石灰岩内流动, 但流量和温度有变异性。地下的 3D 电阻率模型揭示了两个突出的结构:(1) 两个石灰岩岩性之间的 NW 向断层接触; 和 (2) 可能新生代的溶蚀增强的和N向的断层。两个结构的交叉点使石灰岩基岩发生岩溶作用, 形成了通道, 促进了都柏林盆地石灰岩序列内相对较深的热液循环 (估计深度可能在 240 至 1,000 m 之间)作用。通过这项研究发现每年冬天在圣戈尔曼井观察到的最高温度和同时增加的排泄量是结构控制的热液循环系统中大气水快速入渗、加热和再循环的结果。

## Resumo

Um modelo hidrogeológico conceitual da fonte, vias de circulação e variação temporal de uma fonte termal de baixa entalpia em um ambiente de calcário fraturado é derivado de uma abordagem multidisciplinar. O poço de St. Gorman é uma fonte termal no centro-leste da Irlanda com um perfil de temperatura complexo e variável (máximo de 21.8°C). Os dados geofísicos de uma pesquisa áudio-magnetotelúrica (AMT) tridimensional (3D) são combinados com dados hidrogeológicos em intervalos de tempo e informações de uma análise hidroquimica publicada anteriormente, para investigar a operação deste intrigante sistema hidrotérmico. A análise hidroquimica e as medições em intervalos de tempo sugerem que as águas termais fluem de dentro dos calcários fraturados da Bacia Carbonifera de Dublin o tempo todo, mas exibem variabilidade na descarga e na temperatura. O modelo de resistividade elétrica 3D da subsuperfície revelou duas estruturas proeminentes: (1) um contato defeituoso alinhado a NO entre duas litologias de calcário; e (2) uma falha dissolucionalmente aumentada, alinhada a N, de provável idade Cenozóica. A intersecção dessas duas estruturas, que permitiu a carstificação da rocha calcária, criou condutos que facilitam a operação de circulação hidrotérmica relativamente profunda (profundidade estimada entre 240 e 1,000 m) dentro da sucessão de calcário da Bacia Dublin. Os resultados desse estudo suportam a hipótese de que a temperatura máxima e o aumento simultâneo da descarga observada no poço de St. Gorman a cada inverno é o resultado da rápida infiltração, aquecimento e recirculação de águas meteóricas dentro de um sistema de circulação hidrotérmica estruturalmente controlado.

## Introduction

The potential to exploit the geothermal energy of deep, thermal groundwater has been explored in Ireland as part of the IRETHERM project (2011–2016), which was funded by Science Foundation Ireland (grant number 10/IN.1/I3022). A number of low-enthalpy thermal springs were investigated using a multidisciplinary approach, integrating geophysical surveys, time-lapse measurements of hydrogeological parameters, and detailed hydrochemical analysis, with the aims of (1) identifying the source aquifer(s) for the thermal groundwater, (2) characterising the circulatory systems, and (3) assessing the potential for the existence of deeper, higher temperature, circulation patterns for future geothermal exploitation. Two publications have been produced already from this project; Blake et al. (2016a) present the results of a hydrochemical analysis of a set of Irish thermal springs, and Blake et al. (2016b) describe a geophysical survey at one of the springs (Kilbrook spring, Co. Kildare). This paper presents a case study of the most thermally variable of the springs, St. Gorman’s Well, which is located in east-central Ireland in the limestones of the Carboniferous Dublin Basin.

**Fig. 1:**
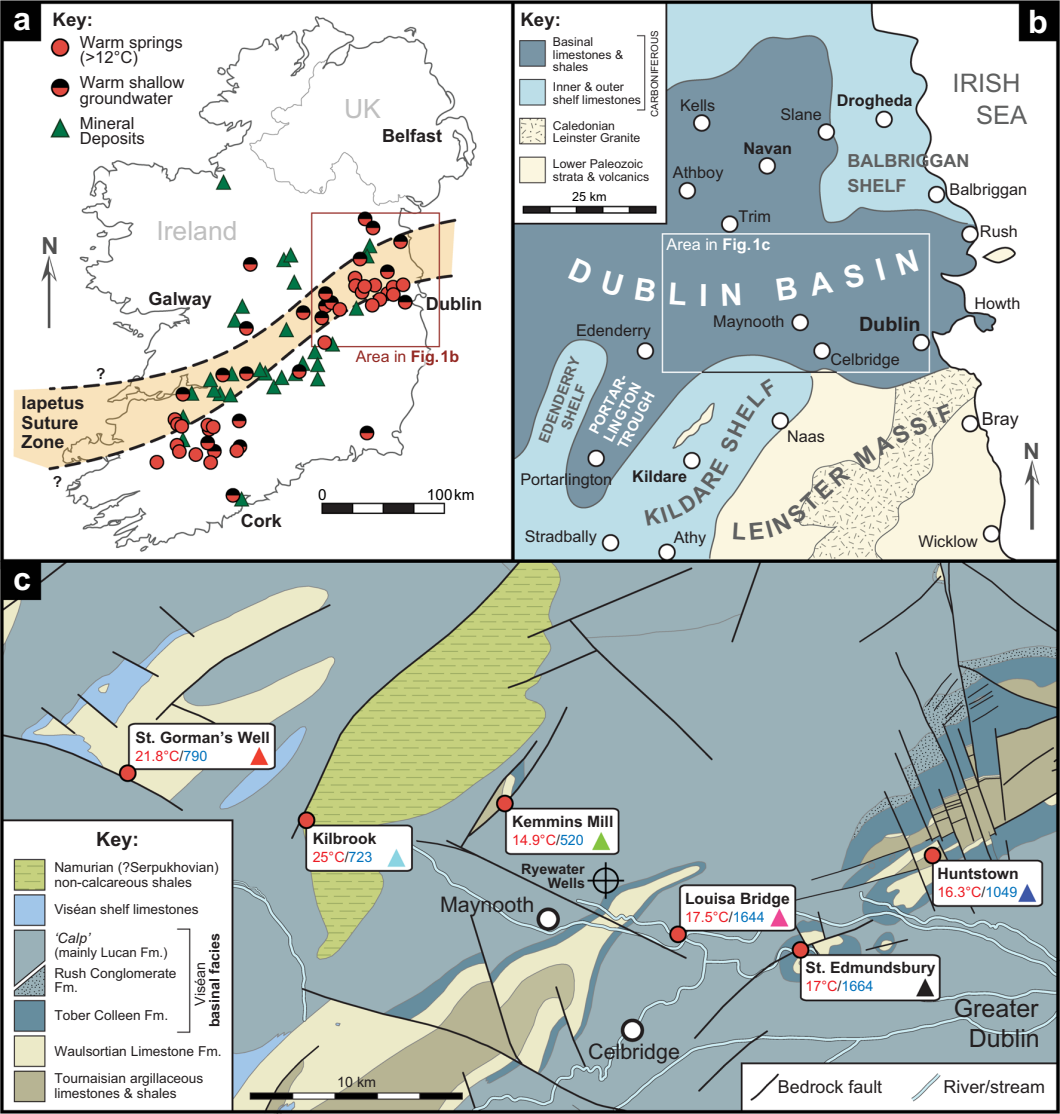
Geological setting of Irish thermal groundwaters: **a** Irish thermal spring and thermal shallow groundwater locations (after Goodman et al. 2004), with mineral deposits and the approximate trace of the Iapetus Suture Zone (after Wilkinson 2010). The area north of the Iapetus Suture Zone represents the former Laurentian landmass, whilst the area to the south of it formed part of the Avalonian continent; **b** paleogeographic map of the Dublin Basin during the Viséan Stage (modified from Sevastopulo and Wyse Jackson 2009); and **c** geological map of the study area (from the Geological Survey of Ireland) showing thermal springs included in the hydrochemical sampling programme. Maximum temperatures (red) and electrical conductivities in μS/cm(blue) are given for each thermal spring (measurements collected during the IRETHERM project). Coloured triangles in each of the thermal spring labels refer to colour coding used for these locations in subsequent figures

Average groundwater temperatures across the island of Ireland typically range from 9.5 to 10.5 °C (Aldwell and Burdon 1980) and thermal springs are considered to be those natural groundwater springs where the mean annual temperature is appreciably warmer than average groundwater temperatures (Aldwell and Burdon 1980; Goodman et al. 2004). St. Gorman’s Well is located close to the urban centre of Enfield, Co. Meath (Fig. 1), and has historical and cultural significance as a “holy well”. There is evidence that this spring was originally dedicated to St. Ultan (Conway 2010), and the current name of the spring probably derives from an anglicisation of the Irish word *goradh*, which means heat. St. Gorman’s Well discharges naturally as an ephemeral pond, and is typically dry during the summer months. Several boreholes were drilled close to the spring in the late 1980s (Murphy and Brück 1989); two of these situated 20 m west of the pond discharge artesian warm waters in the winter (SG4 and SG7). Waters from boreholes SG4 and SG7 exhibit some of the highest shallow groundwater temperatures in Ireland (maximum of 21.8 °C recorded in SG4 during this study). Borehole SG4 was the main monitoring point for this study as this allowed measurements to be taken even when the pond was dry (SG4 is hereafter referred to in this paper as St. Gorman’s Well).

The temperature and discharge profile of St. Gorman’s Well is complex and varies throughout the year. Temperatures are highest in winter, and ranged between 10.5 and 21.8 °C during this study. The maximum discharge occurs in winter (1,018 m^3^/day measured in January 1982 (Burdon 1983; Table 1) with a mean annual discharge of 398 m^3^/day. This variation adds further complexity to the characterisation of the spring as a geothermal resource, as the amount of thermal energy available for exploitation varies throughout the year.

**Table 1:** Monthly discharge data for St. Gorman’s Well pond from 1982

Month	Rainfall (mm)	Discharge (m^3^/day)
Jan-82	84	1017.6
Feb-82	58	616.8
Mar-82	80	746.4
Apr-82	25	549.6
May-82	67	177.6
Jun-82	120	81.6
Jul-82	19	26.4
Aug-82	81	4.8
Sep-82	77	0.0
Oct-82	106	4.8
Nov-82	119	768.0
Dec-82	87	780.0

Reproduced from Burdon (1983). Rainfall data for the same period from Longwood, Co. Meath

This paper presents a three-dimensional(3D) electrical resistivity model from an audio-magnetotelluric(AMT) survey at St. Gorman’s Well. The AMT method is a passive electromagnetic geophysical technique that is widely used for exploring geothermal resources (e.g., Arango et al. 2009; Barcelona et al. 2013; Piña-Varas et al. 2014; Zhang et al. 2015; Blake et al. 2016b) and hydrogeological targets (e.g., Falgàs et al. 2011; Kalscheuer et al. 2015) due to its ability to detect low-resistivity, water-bearing rocks in the subsurface. The depth of penetration can be several hundred metres and even greater than a kilometre below the surface, depending on the resistivity of the bedrock. When used as part of a multidisciplinary approach (incorporating geological, hydrogeological, and other geophysical data), AMT can be an efficient method for improving the characterisation of a fractured rock groundwater circulation system (see, e.g., Blake et al. 2016b). The 3D geophysical model is discussed alongside detailed time-lapse hydrogeological measurements (temperature, electrical conductivity and water level) and a previously published analysis of seasonal hydrochemical data from the spring (Blake et al. 2016a) to investigate and develop a conceptual model of the operation of this unusual thermal spring.

## St. Gorman’s Well in context

### Geology

Irish thermal springs occur in Carboniferous limestone bedrock along a wide band that traverses the centre of Ireland from NE to SW, broadly coincident with the putative trend of the Lower Paleozoic Iapetus Suture Zone (ISZ; Fig. 1a). The ISZ was produced by the final closure of the Iapetus Ocean in late Silurian times, during the later stages of the Caledonian Orogenic cycle (e.g., Chew and Strachan 2014). Following collision, terrestrial sediments were deposited during the Devonian (e.g., Graham 2009), before a shift to predominantly carbonate deposition as a result of a regional marine transgression during earliest Carboniferous (Tournaisian) times (MacDermot and Sevastopulo 1972). During the Tournaisian and Viséan, several intracratonic basins developed across Ireland as a result of tectonism and subsidence (e.g., Strogen et al. 1996; Somerville 2008; de Morton et al. 2015), principally controlled by movement on NE–SW oriented structures, whose orientation was inherited from underlying Caledonian features (Worthington and Walsh 2011; see also Walsh et al. 2019). Extensive carbonate production continued in Ireland for much of the Mississippian, before a switch to terrigenous mud and sand deposition in the Serpukhovian and Bashkirian (formerly regionally termed the Namurian in northwest Europe: see Sevastopulo and Wyse Jackson 2009; Barham et al. 2015 Fallon and Murray 2015).

St. Gorman’s Well is situated in the Carboniferous Dublin Basin (Fig. 1b), which contains circa 2,000 m of sedimentary infill including evidence of the widespread development of carbonate banks or build-ups (‘reefs’) during late Tournaisian to early Viséan times (Somerville et al. 1992). This particular facies, commonly termed the Waulsortian Limestone Formation (Fm.), is characterized by very fine-grained, pure carbonates containing sparry masses. Bedding within the carbonate buildups is often indistinct; these buildups commonly formed aggregates, and intervening off-mound facies are typically represented by thin, nodular, chert-rich shales (Lees and Miller 1995). The relative purity of this carbonate facies results in it being prone to chemical dissolution and the development of karst features, particularly when fractured (Murray and Henry 2018). Active tectonism during the Viséan age led to the development of shallow shelf platforms and contrasting deeper regions in the Dublin Basin. The deeper basinal facies is characterized by thinly interbedded, cherty limestones and shales (mapped regionally as the Lucan Fm., and commonly referred to as “Calp”; see Marchant and Sevastopulo 1980).

St. Gorman’s Well discharges from the Waulsortian Limestone Fm. near to its faulted contact with the younger Lucan Fm. Bedrock exposure in the area is generally poor. The SG8 deep geothermal borehole was drilled very near to St. Gorman’s Well (Murphy and Brück 1989) to a depth of 510 m. The historical data from boreholes close to the spring suggest that: (1) the spring is situated on or very close to a westward dipping, faulted contact between Waulsortian and Calp limestones; (2) significant fracturing and cavities are present in the Waulsortian limestones at depths of 38–55 m in SG4 and SG7, and depths of 68–74 m, 257–263 m, and possibly 510 m in SG8; and (3) the downhole groundwater temperatures peak at 21.7 °C in the cavity zone at depths of 68–74 m in SG8. A recent downhole camera survey of SG4 and SG7 revealed a significant cave or conduit at a depth of 91 m in SG7 (Geological Survey Ireland, unpublished data, 2021). This information suggests that the high groundwater temperatures observed at St. Gorman’s Well are the result of deep circulation patterns, controlled by the presence of permeable structures and conduits within the Waulsortian limestones. Developments of Waulsortian facies in the Dublin Basin can exceed stratigraphic thicknesses of 500 m (Somerville et al. 1992; Strogen et al. 1996), and a thickness in excess of 460 m was recorded for this unit in SG8 (Murphy and Brück 1989).

### Structural geology

The Carboniferous limestones in Ireland that host the thermal springs generally tend to exhibit poor primary porosity. Secondary porosity and permeability are greatly improved by both fracture and karst development, providing discrete pathways for groundwater flow; it is therefore important to consider structural controls on fluid flow within these limestones. In carbonates, the development of deep dissolutional features (at depths of at least 500 m) is likely to be controlled and facilitated by prominent fault structures (Kaufmann et al. 2014). Irish thermal springs are frequently associated with deep-seated, high-angle faults, which facilitate the movement of warm waters towards the surface (Mooney et al. 2010), and they appear to be associated with the dominant, Caledonian, NE–SW structural lineaments apparent in Ireland’s bedrock (Fig. 1a). Gravity surveys in the Leinster region have highlighted the importance of NE-trending alignments in the tectonic fabric of the crust (e.g., O’Reilly et al. 1996), which are prominent due to reactivation of Caledonian thrust faults in the early Carboniferous causing density variations in the Carboniferous cover. Airborne electromagnetic data, recently collected in northern Leinster by Geological Survey Ireland’s Tellus programme (Geological Survey Ireland 2021b) have also highlighted a clear regional NE structural trend. These deep-seated, pervasive faults, although no longer tectonically active, may still provide fluid pathways enhanced by dissolutional processes in discrete zones (through karstification), allowing water to flow from deeper units up to the surface, and are probably very important in controlling regional groundwater flow (Henry 2014). As previously noted, dissolutional features in the Waulsortian limestones in the Dublin Basin near St. Gorman’s Well (Fig. 1c) have been reported at depths of 100–300 m(borehole reports from EMD 2021) and may possibly exist at 510 m in one reported instance (Murphy and Brück 1989). These features play an important role in the operation of deep groundwater circulation patterns and probably facilitate the movement of the thermal spring waters to the surface.

The development of secondary porosity in the Waulsortian Limestone Fm. is likely to contribute to the development of thermal springs in the Dublin Basin, as four out of six thermal springs studied in detail during the IRETHERM project issue from, or have a close spatial association with, mapped surface outcrop of Waulsortian strata, as shown in Fig. 1c). The centres of Waulsortian banks or buildups are typically massive (see Lees and Miller 1995), so any karstic dissolution will tend to exploit areas of fissured and fractured rock (Murray and Henry 2018). By comparison, the chert-rich, off-bank facies are much less soluble, and may thus act to constrain or focus groundwater flow. Flow within discrete Waulsortian mounds can become concentrated along vertical or subvertical pathways with relatively little lateral dissipation of flow (Moore et al. 2015).

**Fig. 2:**
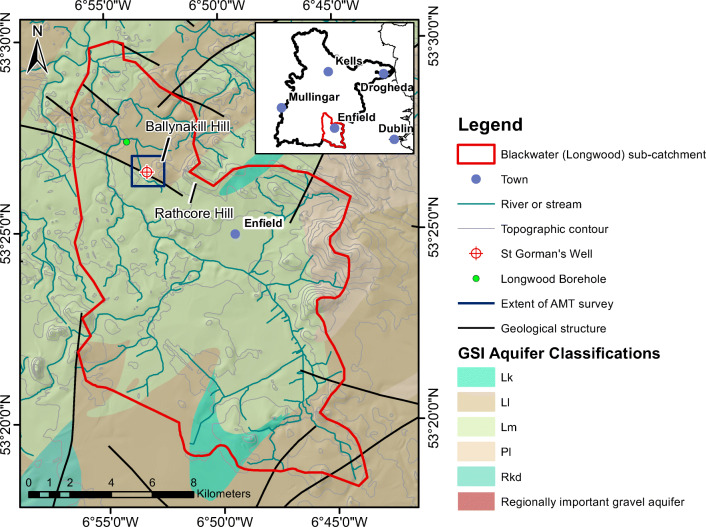
Hydrological environment near St. Gorman’s Well (inset shows the extent of the River Boyne catchment). Topographic contours are in metres above ordnance datum. Aquifers are categorised by Geological Survey Ireland to describe both resource potential and groundwater flow type; Lm, Ll and Lg discussed in the text, for more information see Geological Survey Ireland (2017)

Large Carboniferous faults in the Irish Midlands tend to have an inherited Caledonian alignment (NE–SW) and can be geometrically linked to contemporaneous NW-oriented cross faults, relay faults and splays that controlled Carboniferous basin bathymetry and patterns of sediment deposition throughout the region. These Carboniferous normal faults were subsequently reactivated as thrust faults during later compressional tectonic events (e.g., Hitzman 1999); they act as impermeable barriers to groundwater flow because of incorporated host-rock clays and shales created by a combination of fault rock attenuation and smearing, and by dissolution-related restite formation (Moore and Walsh 2013). Carboniferous and Variscan structures in the Irish Midlands are offset by NW- and NE-oriented conjugate Cenozoic strike-slip faults, which are the main groundwater-controlling structures in quarries and mines in the region, and can be identified by their distinct iron-oxide staining due to groundwater flow (Moore and Walsh 2013). The Cenozoic strike-slip faults were formed by subhorizontal N–S Alpine compression (Cooper et al. 2012), under low confining pressures, and have been identified in Irish mines at depths in excess of a kilometre (Moore et al. 2015). In certain locations, particularly where they are intersected by Cenozoic strike-slip faults, the Carboniferous normal faults can become karstified and have their permeability greatly increased (Moore and Walsh 2013). A local example of such an intersection of structures can be seen at Rathcore Quarry, 1.5 km east of St. Gorman’s Well (Fig. 2). Here, the intersection of a Carboniferous normal fault and a N-oriented Cenozoic strike-slip fault has resulted in the development of a large karstic depression (20 m wide), which has been subsequently filled with unconsolidated materials.

St. Gorman’s Well is situated on a normal-fault-bounded inlier of Waulsortian Limestone Fm. in the western part of the Dublin Basin. A NW-oriented, normal fault is mapped close to St. Gorman’s Well, juxtaposing downthrown younger Lucan Fm. sediments to the west (hanging wall) and upthrown older Waulsortian Limestone Fm. to the east (foot wall). On the basis of borehole records, Murphy and Brück (1989) estimated the dip of the fault to be approximately 60 ° W. The fault has a minimum vertical displacement of 750 m and is likely to be deep-seated and of Carboniferous age.

### Hydrogeology

**Fig. 3:**
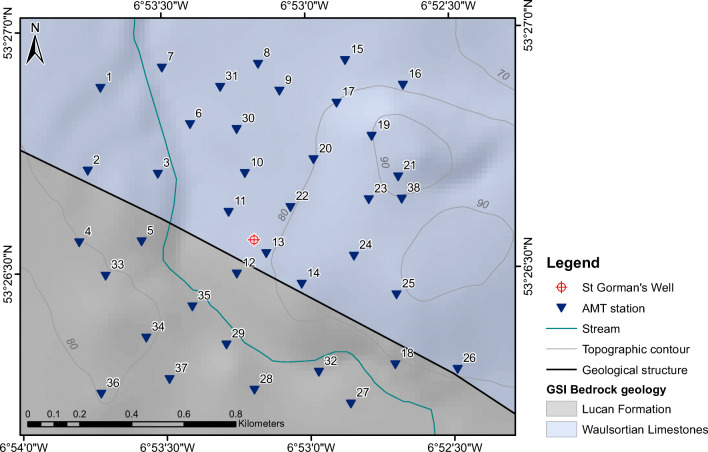
AMT station locations and local geology (from the Geological Survey of Ireland) at St. Gorman’s Well. The Lucan Formation contains Viséan basinal limestones, and is also called ‘Calp’. Topographic contours are in metres above ordnance datum

St. Gorman’s Well is situated 40 km west of Dublin, between the towns of Longwood and Enfield in Co. Meath, in a relatively flat and low-lying landscape in the catchment of the River Boyne (Fig. 2). The spring is located in the River Blackwater (Longwood) subcatchment and is designated as a Priority Area for Action (PAA) in the National River Basin Management Plan 2018–2021. The elevation in the geophysical survey area in Fig. 3 ranges from approx. 70 to 90 m above ordnance datum, and the spring is situated at an elevation of 80 mAOD. The land rises gently across the survey area from west to east and peaks locally at Ballynakill hill (94 mAOD) 600 m NE of the spring, and Rathcore quarry (123 mAOD) 2 km east of the spring (Fig. 2). The main use of land is agricultural, with very limited bedrock exposure, and the spring and its surroundings have been proposed as a National Heritage Area by Geological Survey Ireland.

A moderately dense network of streams flows from southeast to northwest to drain the subcatchment and the discharge point is located some 6 km NW of St. Gorman’s Well. Many of the smaller streams in the vicinity of the well are thought to be groundwater-fed due to their chemistry (Dillon and Kelly 2010). The 30-year (1981–2010) average annual rainfall in the area is 868 mm/year(Walsh 2012); during the sampling period (for the hydrochemical sampling and time-lapse measurements) the annual rainfall was 863 mm in 2013 and 922 mm in 2014 (data from Met Éireann 2021). Evaporative losses for the region are estimated at 450 mm/year(data from Met Éireann 2021). Most recharge to aquifers in Ireland occurs in the period between October and April, and typical estimated recharge rates for the area surrounding St. Gorman’s Well are between 200 and 293 mm/year (Geological Survey Ireland’s groundwater recharge maps, Geological Survey Ireland 2021a). Geological Survey Ireland’s “Groundwater 3D” project has developed new hydrogeological conceptual models for the Boyne catchment (Geological Survey Ireland, unpublished report, 2021). St. Gorman’s Well is situated in the “South Eastern zone” (Fig. 2), which demonstrates the highest potential for developing new groundwater resources within the Boyne catchment. In this zone, groundwater flow is dominant over surface water flow and it is estimated that between 41 and 72% of the effective rainfall is recharging.

Depth to bedrock at the spring is 3 m and the subsoil consists of limestone till (Burdon 1983) and possibly some gravels derived from limestones (Dillon and Kelly 2010). The Lucan Fm., or Calp, is categorised by Geological Survey Ireland as a “locally important, generally moderately productive (Lm)” aquifer, and the Waulsortian Limestone Fm. in the area is categorised as “locally important, moderately productive in local zones only (Ll)”. Significant and productive local gravel (Lg) aquifers are present near Longwood, to the northwest of the spring (Fig. 2). It is worth noting that Geological Survey Ireland usually considers an effective lower depth limit of 150 m for Irish aquifers (Fitzsimons et al. 2005). Hydraulic data are available from a public water supply borehole at Longwood, which abstracts approximately 350 m^3^/day from a single borehole in gravels and fractured limestones. There the gravels have an apparent transmissivity of 80 m^2^/day (it is noted that these saturated gravels are unlikely to be laterally extensive over a large area) and flow in the bedrock is concentrated in the upper fractured and weathered zone (Dillon and Kelly 2010).

The pond at St. Gorman’s Well is typically filled during winter, in the high recharge period (October–April), when its surface area is approximately 40 m^2^. In the summer, the artesian discharge from the boreholes SG4 and SG7 decreases and the pond is typically dry by September (Table 1). As mentioned previously, samples and measurements were taken from SG4 for this study. No detailed borehole logs could be obtained for SG4 but some limited information is available in Murphy and Brück (1989). SG4 is a 48 m deep, open hole in the limestone bedrock that is cased to a depth of 5.8 mbgl. The borehole has its maximum discharge in January, when the water level is artesian; this is when the water temperature is at its maximum (21.8 °C recorded during this study). Subsequent to the study period, in winter 2016–2017, the spring did not become artesian due to an unusually dry autumn and winter period in that particular year. The borehole discharges to a land drain, which joins a small stream 0.8 km northwest of the borehole. This small stream flows northward and eventually joins up with the River Blackwater near Longwood. The catchment area of the Blackwater at Castlerickard (station 7003), immediately north of Longwood has been determined to be 181.5 km^2^(Office of Public Works 2021) and the regional groundwater flow direction is estimated to be from the southeast to the northwest (Dillon and Kelly 2010), coincident with that of the River Blackwater. It is feasible that the spring could receive shallow recharge from the nearby local peaks at Ballynakill and Rathcore (Fig. 2), where the glacial till subsoil covering the Waulsortian limestones is thin. For the duration of this investigation (2013–2015), the maximum winter discharge from the borehole was estimated to be approximately 1,000 m^3^/day; this is concurrent with the monthly discharge measurements for the spring pond in 1982 (Table 1). These historical discharge measurements indicate that the spring has a mean annual discharge of approximately 400 m^3^/day. A simplistic water mass balance was carried out to assess a possible recharge area to St. Gorman’s Well during its season of maximum discharge—Table S3 in the electronic supplementary material (ESM). Assuming all of the groundwater comes from a karst limestone aquifer in the Waulsortian Limestone Fm. with an effective porosity of 0.01—this value is based upon lower end of range of values for regional karstic aquifers (OCM 2010)—the thermal spring has a contributory area of 145 km^2^, which could feasibly be contained within the Blackwater (Longwood) subcatchment (area of 181 km^2^).

The water from St. Gorman’s Well has a Ca-HCO_3_-type hydrochemical signature, which is typical of many recently infiltrated, cold, Irish groundwaters circulating in limestones, and also typical of the majority of the Irish thermal springs. The hydrochemical signatures of the thermal springs imply that they are mainly composed of meteoric waters that are recently recharged from rainfall events (Burdon 1983; Mooney et al. 2010). Burdon (1983) showed that St. Gorman’s Well contained lower tritium levels than cold groundwater (samples collected in August 1982). These low tritium levels, along with the elevated temperatures, are suggestive of longer residence times and deeper circulation patterns for the thermal groundwater. Water from St. Gorman’s Well is likely to be a blend of groundwaters from different sources and different recharge areas. The thermal water could be composed of a mixture of a deeper-circulating, older groundwater, and more recent, meteoric recharge water from a shallow groundwater system.

### Previous hydrochemical analysis

This section discusses hydrochemistry results and interpretations relating to St. Gorman’s Well that were previously published in Blake et al. (2016a). During the course of the IRETHERM project, samples were collected and analysed from the six thermal springs in Fig. 1c). Geographical coordinates, geological setting, maximum temperatures and a brief description of each spring is provided (ESM). Data were recovered for analysis over five seasons to assess the temporal variation in the spring chemistry and to provide some seasonal overlap for a more robust analysis. The springs were sampled in July/August and October 2013, and in January, May and August 2014.

**Fig. 4:**
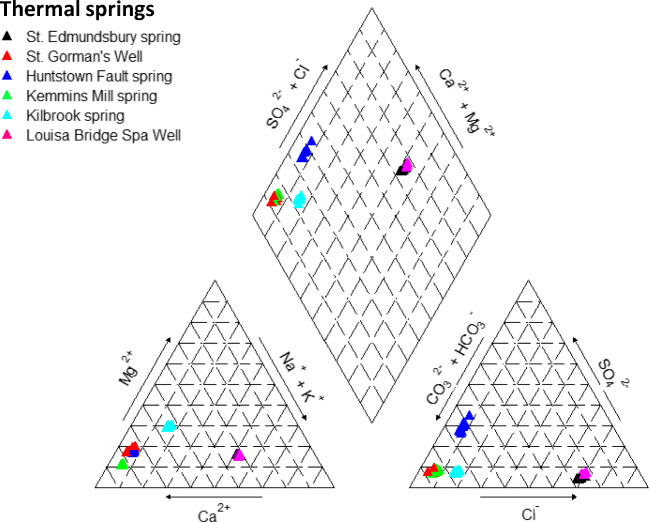
Piper diagram of hydrochemical analyses from the Leinster thermal springs including St. Gorman’s Well

The major ion chemistry of St. Gorman’s Well, as measured during the sampling period from July 2013 to August 2014, is comparable to Irish Ca-HCO_3_-type groundwaters and reflects the findings of previous studies (e.g., Burdon 1983). It is clear from Fig. 4 that the major ion hydrochemistry of St. Gorman’s Well varies little throughout the year, despite its large variations in temperature. Blake et al. (2016a) describe the statistical analysis of the hydrochemical dataset; this analysis revealed nothing in the hydrochemistry of St. Gorman’s Well to suggest the presence or influence of deep-basinal fluids. In other words, the major ion chemistry of St. Gorman’s Well is typical of Ca-HCO_3_-type groundwater at all times, even when the temperatures and discharges are simultaneously at their maxima. A compositional multivariate statistical analysis (principal component analysis) was used to uncover patterns and associations between hydrochemical variables in the dataset as a whole (Blake et al. 2016a). The chemistry of St. Gorman’s Well was distinguished from other Ca-HCO_3_-type groundwaters in the dataset as having a stronger association with Si and HCO_3_ and a weaker association with SO_4_; this was interpreted as the influence of the dissolution of the pure carbonate and associated chert layers of the Waulsortian facies on St. Gorman’s Well. The statistical analysis also revealed that St. Gorman’s Well has the largest relative temporal variations in trace element hydrochemistry of all the thermal springs sampled. The concentration of dissolved metals (Mn and Co in particular) in the groundwater increases during the summer (May to October); this increase in dissolved material is reflected by a general increase in electrical conductivity during the same period. This seasonal difference could suggest that the winter thermal water system has a different, and perhaps less evolved, hydrochemistry to the summer thermal water system due to greater dilution with fresh recharge waters in winter.

## Methodology

The methodology followed in this investigation is similar to that employed by Blake et al. (2016b) at Kilbrook spring (see Fig. 1), where the AMT method, geophysical survey, data processing and modelling procedures for Kilbrook Spring are discussed in detail. For this study of St. Gorman’s Well, the data processing and modelling procedures are discussed briefly, with further details provided in the ESM.

### AMT survey

The AMT method determines the distribution of the electrical properties of the subsurface and the results can be expressed in terms of either electrical conductivity (S/m) or electrical resistivity (Ωm). Here, the results of the AMT survey are expressed in terms of resistivity.

The magnetotelluric (MT) method is a geophysical technique that determines the distribution of electrical resistivity in the subsurface by relating simultaneous measurements of the naturally occurring fluctuations of the electric and magnetic fields at the Earth’s surface. Recent comprehensive reviews of the MT method are provided by Simpson and Bahr (2005), and Chave and Jones (2012). Natural electromagnetic fields that are utilised as source fields in MT studies range in frequency from approximately 10^−5^ to 10^5^ Hz. Audio-magnetotelluric(AMT) studies utilise higher frequency (>8 Hz) electromagnetic waves that are generated by electric lightning discharge during lightning storms and propagate around the globe in the Earth-ionosphere waveguide.

The AMT survey was designed to target any karstic conduits occurring beneath St. Gorman’s Well. Thirty-eight AMT measurement locations (stations) were laid out in an approximate grid pattern, centred on the spring itself, with approximately 200 m between sites (Fig. 3). The grid covered a total area of 2.6 km^2^. This layout was chosen to investigate depths in excess of 100 m beneath the spring (with a separation of 200 m between stations at the surface, the volumes of measurement beneath each of the stations first overlap at a depth of around 100 m, thus providing a more reliable estimation of the properties of the subsurface at depths greater than 100 m). The survey was carried out in October 2013 to take advantage of cleared fields that had been harvested. Overnight AMT measurements were made using Phoenix MTU-5 systems with an electrode array and horizontal magnetic coil configuration oriented to geomagnetic north–south–east–west, combined with a vertical magnetic recording at each station. Data were acquired in the frequency range between 1 and 10,000 Hz. As the data quality in populated areas is often affected by man-made (“cultural”) electrical noise, one system was deployed as a remote magnetic reference station in a culturally quiet location approximately 9.5 km north of the spring. This extra station allowed for remote reference processing (Gamble et al. 1979). The AMT time series were processed using Phoenix SSMT2000 software, which employs a robust variant of a remote reference processing algorithm based on Jones and Jödicke (1984), and Jones et al. (1989). Aside from the AMT dead-band (a frequency interval with poor signal-to-noise ratio that is found between 1,000 and 5,000 Hz), the data quality was generally good between 10 Hz and 10,000 Hz. The impedance tensors (**Z**) and the vertical magnetic transfer functions (**T**) were estimated for each frequency for each station. Each curve was manually edited to remove excessively noisy data in the AMT dead-band).

**Fig. 5:**
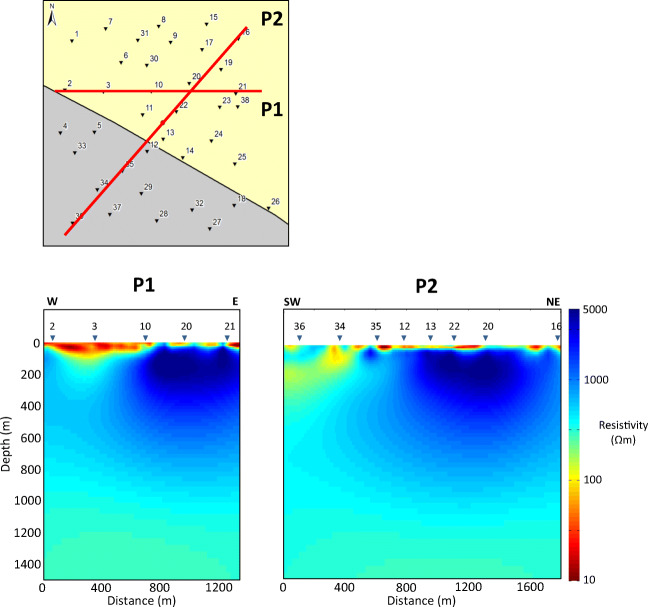
Vertical profiles (P1 and P2) through the final resistivity model. The profile locations are indicated in the plan view of the survey area. The locations of the AMT stations are indicated by inverted triangles

**Fig. 6:**
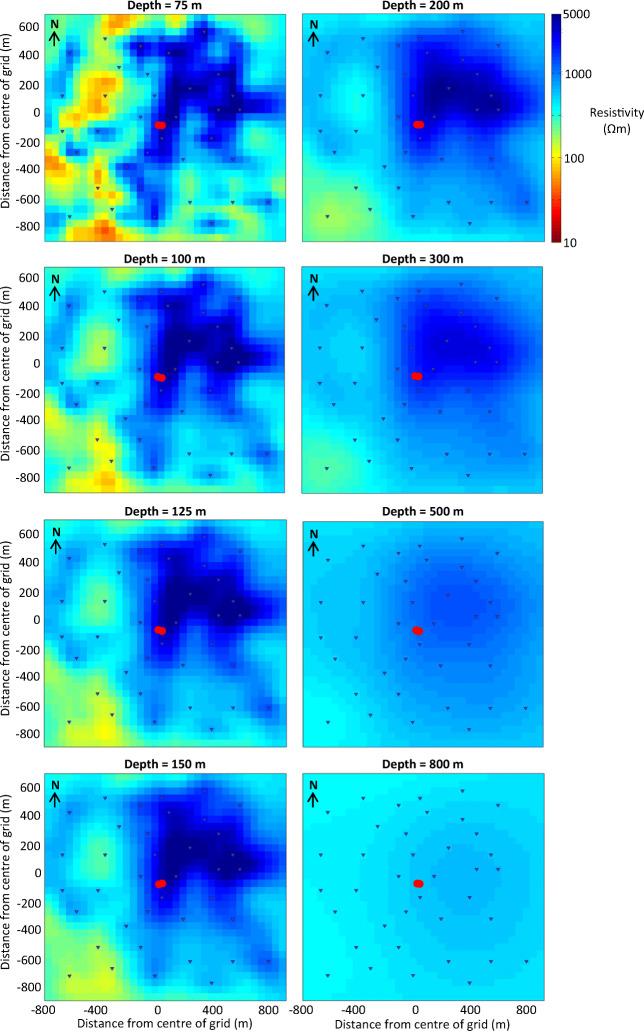
Horizontal slices through the final 3D resistivity model. The depth of each slice is indicated. The area of each plot corresponds to the extent of the survey area indicated in Figs. 2 and 3. The spring is located at 53°26′34.57″N 6°53′9.68″W

The dimensionality of the data was analysed by investigating the **Z** and **T** responses independently of each other (section S2.1 of the ESM), and the results indicate the existence of a 3D scenario beneath the survey area. A 3D inversion was adopted as the most appropriate course of action. The data were inverted using the ModEM 3D inversion code (Egbert and Kelbert 2012; Kelbert et al. 2014) (Figs. 5 and 6), and further details of the inversion process are provided in the ESM (section S2.2).

### Time-lapse hydrogeological measurements

**Fig. 7:**
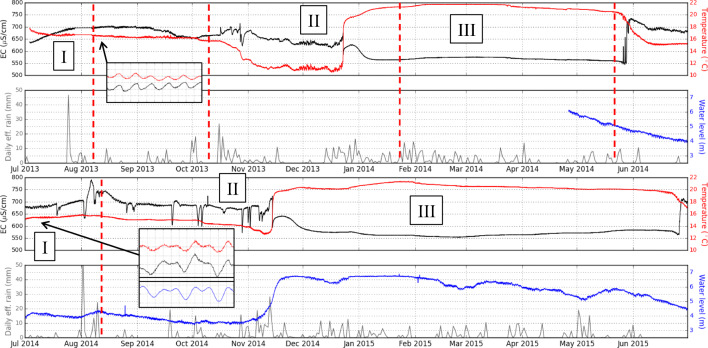
Time-lapse temperature (red), electrical conductivity (black), and water level (blue) measurements for St. Gorman’s Well. Water level is presented in metres above an arbitrary datum. Daily effective rainfall data (grey) is from the Met Éireann synoptic station in Dunsany, Co. Meath. First two panels show data from 2013 to 2014; second two panels show data from 2014 to 2015. Insets for August 2013 and July 2014 show 3 days of semidiurnal fluctuations in temperature and EC (and water level for 2014 only). Dashed red lines indicate hydrochemical sampling rounds. Roman numerals indicate interacting hydrogeological flow systems. Dashed black arrows indicate sudden switch from flow system II to III in 2014, and corresponding water level

Continuous temperature and electrical conductivity (EC) measurements were collected at St. Gorman’s Well between July 2013 and July 2015, and are presented along with effective rainfall data in Fig. 7. Daily rainfall and potential evapotranspiration data from Dunsany synoptic station (data from Met Éireann 2021), situated 14 km to the northeast of the spring, were used to estimate the effective rainfall. Some of this data featured in Blake et al. (2016a), but the data from late April 2014 to July 2015 are presented here for the first time. Water level measurements were also recorded from late April 2014 to July 2015. Further details of the logger installation are provided in section S1.1 of the ESM. Summary statistics for the data were calculated using Onset HOBOWare® software (version 3.4.1; Table 2).

**Table 2:** Summary physico-chemical statistics for St. Gorman’s Well

pH range	Max EC (μS/cm)	Min EC (μS/cm)	Mean EC (μS/cm)	Max T (°C)	Min T (°C)	Mean T (°C)	SD T (°C)
6.7–7.8	790	544	631	21.8	10.5	17.7	3.0

Temperature (*T*) and electrical conductivity (*EC*) data from logger measurements. pH measured in field with Hanna Combo meter during data collection rounds.* SD* standard deviation

## Results and discussion

In this section, results from the 3D AMT survey, time-lapse hydrogeological logger data, and hydrochemical interpretations from Blake et al. (2016a) are presented and discussed to investigate the operation of the hydrothermal system at St. Gorman’s Well.

### Final 3D AMT model

The published bedrock geological map of the region (McConnell et al. 2001) indicates the presence of limestone throughout the survey area (Figs. 1 and 3). The resistivity values of limestone depend upon a variety of factors such as clay content and porosity. Unweathered limestone can generally have high resistivity values of between 1,000 and 100,000 Ωm (between 10 and 0.1 μS/cm); however, shale horizons can reduce the bulk resistivity to values as low as 10 Ωm (1,000 μS/cm; Palacky 1987). The amount of fluid contained in the rock will also reduce its bulk resistivity (Telford et al. 1990). Seawater has a low resistivity of less than 1 Ωm (10,000 μS/cm), whereas freshwater has higher resistivities of up to 100 Ωm (100 μS/cm) (Palacky 1987).

Even in heavily karstified regions, large cavities in limestones (caves and fissures) tend to range in size up to 10 m (Kaufmann et al. 2014), and the cavities formed in the limestones of the Dublin Basin are not expected to exceed widths of a few metres. Cavities from the SG8 borehole next to St. Gorman’s Well range in size up to 3.8 m (Murphy and Brück 1989). Given the size of the cells in the AMT model mesh (50 m × 50 m in the central region of interest), the resolution is unlikely to resolve the details of the water-bearing conduits precisely. However, the presence of water-bearing conduits in a volume of limestone bedrock will reduce the bulk resistivity of the rock as a whole, and this should be evident in the model. Faults can also contain clays as well as fluids that reduce the bulk resistivity of the model in the region of the fault. It is noted that the AMT survey was carried out in October 2013, when regional groundwater levels were probably at their lowest.

The final resistivity model converged after 60 iterations with a normalised root mean square (nRMS) misfit of 1.93 (Fig. S3 of the ESM shows the residual misfit of the data to the model responses for each period and each station). Upon examination of the model, and given that the space between stations is approximately 200 m, the model results are more reliable from approximately 100 m depth. Resolution of fine structure decreases with depth, and is best resolved between depths of 100 and 500 m. There is a conductive region in the model between depths of 1,200 and 1,700 m, with resistivity values that are lower than the initial model. Beneath this conductive horizon, the values are the same as the initial model. This conductive region signifies the absolute extent of the sensitivity of the data to variations in resistivity. Although the resolution is too poor to allow this boundary to be pinpointed precisely, this conductor exists, and could potentially represent the limit of the Dublin Basin and the top of the (relatively electrically conductive) metamorphic Ordovician and Silurian basement rocks.

The model shows a large region of high resistivity in the centre and NE of the survey area with the spring located at its centre (Fig. 6). There is a highly resistive core to this region with apparent resistivity values in excess of 5,000 Ωm. There is a smaller region of low resistivity material in the SW corner of the survey area, with minimum resistivities lower than 100 Ωm. The boundary between the low resistivity and high resistivity regions is a relatively sharp, linear feature, which is oriented NW–SE. This boundary appears to have a dip of approximately 40° to the SW when viewed in profile (Fig. 5). Between this boundary and the spring, there are pockets of lower resistivity material (100–1,000 Ωm), which appear to have the same orientation as the boundary, from NW–SE. The largest of these pockets, to the NW of the spring, is a quasiconical body that is 300 m wide and 300 m long at a depth of 100 m below the surface, which extends to a depth of approximately 250 m (the centre of this body is roughly located beneath station 3 in Fig. 5). The sides of this body are oriented N–S, and it aligns with a low-resistivity bulge in the boundary between the low resistivity and high resistivity regions.

**Fig. 8:**
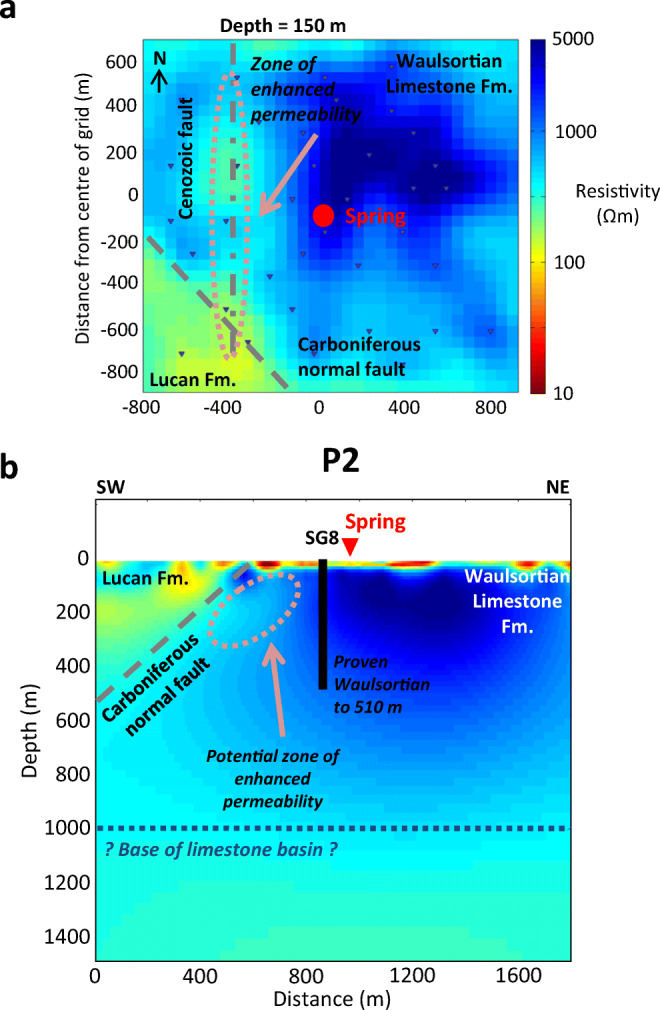
**a** Horizontal slice through the final resistivity model at 150 m depth showing the main intersecting geological structures and potential water-bearing zones of karstified bedrock**. b** Vertical profile through the model (P2 in Fig. 5) showing main features of the hydrothermal flow system. SG8 indicates the extent of the 500-m geothermal borehole from Murphy and Brück (1989)

The main features of the interpreted geophysical model are highlighted in Fig. 8. The results from the model are generally consistent with the published geological map of the survey area. The resistive region in the NE of the model is interpreted as the Waulsortian Limestone Fm., which is expected to be resistive due to the purity of the carbonate and its crystalline nature. The less resistive region in the SW corner of the model is interpreted as the Lucan Fm., which is expected to have a lower resistivity than the Waulsortian Limestone Fm. due to its higher clay content and shale-rich nature. The contact between these two units dips to the SW at approximately 40°, which is reasonably comparable to the dip estimates of Murphy and Brück (1989). This model shows that the contact between the two lithologies lies further south than the contact on the geological map (Fig. 3), and also has a slightly different orientation (the fault on the map is oriented WNW). The vertical extent of the resistive Waulsortian Limestone Fm. appears to be approximately 800 m, although the model loses resolution with depth. The 3D model is in agreement with records from the borehole SG8, which reached a final depth of 510 m in Waulsortian limestones (Fig. 8).

The spring is situated on the SW edge of the zone of highest resistivity within the Waulsortian limestones. Between the spring and the boundary with the Lucan Fm., there is a mottled zone with pockets of lower resistivity material trending from NW to SE in the same alignment as the faulted contact, which probably represents a water-bearing zone of higher permeability. It is likely that the highly resistive Waulsortian limestones have become karstified due to their proximity to the normal faulted contact.

The large, 250 m deep, area of low resistivity, to the NW of the spring, could represent a karstic depression that has subsequently been filled with sediments of lower resistivity (e.g., tills from the last glacial maximum) that are possibly also permeable. The sides of the depression are oriented N–S and the depression itself is directly north of a significant bulge in the faulted contact (Fig. 8). This configuration could represent the intersection of a Cenozoic strike-slip fault and a Carboniferous normal fault and subsequent karst development of high permeability zones along the structures. A large, infilled, karstic depression has been observed 1.5 km east of the spring (see section ‘Structural geology’) where a Carboniferous normal fault is intersected by a Cenozoic strike-slip fault. Cenozoic strike-slip faults in particular are known to produce very high flow rates in other locations in the Dublin Basin (e.g., Huntstown thermal spring, Fig. 1c). Borehole evidence from Murphy and Brück (1989) suggest that in boreholes adjacent the spring, maximum temperatures were encountered in cavities in Waulsortian limestones at a depth of 70 m. A slice through the AMT model at 75 m depth (Fig. 6) shows a linear area of reduced resistivity trending NE–SW from the faulted contact to the spring. This is possibly the feeder conduit for the thermal waters, although the size of the cells in the model mesh are probably too large to resolve the conduit accurately. The resistivity model is more accurate at depths in excess of 100 m due to the spacing between receivers. It may be possible to further develop the conceptual model by carrying out a shallow geophysical survey to resolve the top 100 m (e.g., by using the DC resistivity method).

### Time-lapse hydrogeological measurements

The spring exhibits a distinctive pattern of high-temperature, high-volume, winter discharges and intermediate-temperature, steady, summer behaviour (Fig. 7). This pattern is repeated annually with slight differences; this suggests that the hydrothermal circulation pattern is controlled by annual recharge, and that the spring has a direct hydraulic connection to meteoric recharge. The summer period is characterised by a stable, intermediate temperature (approximately 16 °C) and increased EC (approximately 700 μS/cm) in both years, although the EC in the second year shows a more flashy profile with localised peaks. The onset of the winter recharge period in October is marked by a decrease in temperature (a minimum temperature of 10.5 °C was recorded in 2013, and 12.6 °C was recorded in 2014). The minimum temperatures reached in 2013 are similar to average cold groundwater temperatures. In both years, there is a sudden increase in temperature and simultaneous decrease in EC at a point early in the winter recharge period. This increase in temperature occurred in December 2013, and in November 2014. On December 23rd 2013, the temperature of the spring increased by 6 °C in 6 h. On November 14, 2014, the temperature increased by 4 °C in 12 h. In both years, after this sudden increase, there was a more steady increase in temperature until the spring reached its annual maximum (these maxima occurred in February 2014 (21.8 °C) and January 2015 (21.4 °C). During these periods of sustained high temperatures, steadily low EC values were observed (values between 550 and 600 μS/cm in both years). In the summer period of each year, the temperature of the spring falls back quite rapidly to intermediate temperatures, while the EC has a sudden increase to approximately 700 μS/cm, thus completing the annual cycle.

It appears that the spring responds in a nonlinear way to large inputs of recharge waters to the system. The decrease in temperature after the onset of the winter recharge period in October occurs after the first period of heavy rainfall of the season. In both years, there is a sudden increase in the temperature of the spring a few days after local maxima in the rainfall records (intense rainfall occurred on December 18, 2013 and November 13, 2014). The difference between the temperature profiles for the 2 years is probably due to the amount of recharge input to the system. In winter 2013–2014, the profile is a little more extreme than in the subsequent year, with lower minima and slightly higher maxima. The switch from low to high temperatures in December 2013 is faster and the high temperatures are also more stable throughout the winter recharge period. The total rainfall for the period between October and April (inclusive) is 614 mm for the first year and 518 mm for the second year; this seems to suggest that higher recharge input to the system can result in higher and more sustained winter temperatures for the spring. It is interesting that during the drier summer period, particularly in the first year, localised increases in rainfall do not appear to have any effect on the temperature of the spring.

The water level records began in late April 2014. The water level profile generally follows the same pattern as the temperature profile; i.e., when the water level is increasing, so is the temperature. The maximum water level measured is 6.7 m; this represents the height of the water column above the logger when the flow is artesian in the winter. The transition from intermediate/cold temperatures to high temperatures, which is marked by a sudden change in the temperature and EC profiles, corresponds to a more gradual increasing trend for the water level. The water level decouples from temperature in November 2014 and begins to rise while the temperature continues to fall; this marks a change in the behaviour of the spring as the effects of the warm aquifer are obscured by the rising local and regional groundwater levels with the onset of winter. It can be surmised that the local groundwater level must reach a critical threshold before the temperature increases rapidly (the temperature increase occurred in 2014 when the water level was 5.05 m above the datum, or 1.65 m below the surface). However, both the water level and the temperature reach their maxima around the same time.

The temperature, EC and water level measurements exhibit semidiurnal fluctuations (see insets in Fig. 7), which are more pronounced in the summer period, when water levels are low. Semidiurnal fluctuations in water level for St. Gorman’s Well were identified by Burdon (1983) and compared to gravity tide correction data for a period in 1981. The close correlation of the two signals confirmed the strong influence of the Earth’s gravity tides upon the water levels in the spring, with maximum variations occurring around the times of the new and the full moon (this effect is also observed at Kilbrook spring (Blake et al. 2016b). The relative movements of the Earth, Sun and Moon cause a periodic distortion in the shape of the Earth that causes groundwater to be expelled from aquifers; this is often termed the tidal loading effect and is most measurable in aquifers in rigid, fractured rocks (e.g., Bodvarsson 1970; Rojstaczer and Agnew 1989; Maréchal et al. 2002; Lai et al. 2013). The presence of these semidiurnal fluctuations in water level (Burdon 1983) and temperature (this study) is evidence that both water level and temperature are influenced by tidal forces, and that the thermal groundwater is stored in a fractured, hard-rock aquifer. The prominence of the fluctuations in summer is evidence that the intermediate-temperature thermal groundwater is stored under confined or semiconfined bedrock aquifer conditions during the summer. It is possible that the groundwater is still subject to confined aquifer conditions during the winter, but the artesian flow of the spring dampens and sometimes obscures the semidiurnal signal. Data from this study show that the water level fluctuations mirror those of the temperature and EC (i.e., when temperature and EC are at a local maximum, the water level is at a local maximum; Fig. 7). This implies that the water being periodically expelled from the confined aquifer by the action of the gravity tides is warmer with a higher EC, and this water is being mixed with cooler, less evolved waters, probably in a closed system. The nature of this water is in contrast to the high-temperature, winter waters, which have a lower EC.

The unusual, rapid onset of the high-temperature phase is suggestive of nonlinear, piston flow in karstic conduits. Periodic and intermittent springs are a well-documented feature of some karst aquifers (e.g., a periodic, nonlinear flow pattern was identified in a karstic spring in Derbyshire, England, and attributed to an unstable sediment blockage in the system (Bottrell and Gunn 1991). The lower EC values in winter could be suggestive of dilution by fresh recharge waters. However, the solubility of calcium carbonate is strongly influenced by the presence and availability of CO_2_. It is possible that the thermal component that comes to the surface in winter is stored in an aquifer with little influx of “fresh” CO_2_-carrying waters (e.g., a deep aquifer with a large catchment area and long residence times). An increase in temperature decreases the solubility of CO_2_ and CaCO_3_, along with other carbonates and sulfates, resulting in a lower EC (Gunn et al. 2006). In this way, a low EC may not necessarily indicate a younger recharge water, and the residence times of the thermal waters at St. Gorman’s Well may be long.

St. Gorman’s Well is temporally variable, and its nonlinear response to rainfall suggests a karstic conduit system of transport for the thermal waters. Given the geological setting of the spring it is feasible to envisage a network of karstic conduits interacting at different times of the year, under the influence of seasonal recharge conditions. Figure 7 identifies three distinct hydrogeological flow systems in operation at different times of the year. Each of these flow systems represents a groundwater with a distinct chemistry and temperature pattern.

- System I is steady: it has an intermediate temperature (approximately 16 °C) and likely has a confined or semiconfined deep warm source that mixes with shallower, cooler groundwater.
- System II is cooler: it occurs at the beginning of the winter recharge season when the influence of cooler waters becomes stronger and overwhelms the steady, intermediate temperatures.
- System III is warm: it has a high, steady temperature (>20 °C) and slightly lower (and more stable) EC; the flow is artesian with a discharge of approximately 1,000 m^3^/day, and semidiurnal fluctuations in temperature are largely obscured by the artesian flow.

The switch-over between systems II and III occurs early in the winter, and happens suddenly and nonlinearly; this strongly suggests the presence of conduit flow in karst as the mechanism by which these flow patterns occur. The switch-over from system III back to system I is more gradual, and the temperature decreases as the drier summer season progresses.

### Previous hydrochemical analysis (Blake et al. 2016a)

The year-round Ca-HCO_3_-type signature of the waters suggest that the hydrothermal circulation pattern occurs exclusively in limestone bedrock, i.e., within the Carboniferous Dublin Basin. The seasonal differences in trace element hydrochemistry (an increase in dissolved metals in the summer) indicate that the high-temperature thermal waters of system III (Fig. 7) have a different hydrochemistry to the intermediate-temperature system I. These seasonal differences could be due to the influx of recharge waters to the karstic flow system, which facilitates the operation of a relatively deep circulation pattern within the limestone succession. Two possible explanations for the intricate temporal pattern of St. Gorman’s Well are posited here: (1) cool, dilute, winter recharge waters infiltrate quickly to depth, become heated and mixed, and then rapidly ascend to the surface where they issue with a high temperature in excess of 20 °C and a low EC; or (2) recharge waters infiltrate slowly over a large catchment area and are stored at depth in a thermal aquifer with limited CO_2_ availability, which limits the solubility of carbonates and sulfates in the aquifer and provides a high temperature, yet low EC spring water. In the absence of data on the amount of dissolved CO_2_ in the spring, and due to the strong temporal relationship between rainfall and recharge patterns and the spring’s discharge, it is perhaps more plausible at this time to conceptualise the first option in the preceding, with infiltration of seasonal winter recharge triggering mixing and piston flow of warmer waters.

## Conceptual model

Information from several different strands of enquiry converge on the consensus that the hydrothermal circulation pattern beneath St. Gorman’s Well is governed by the availability of fresh recharge waters and structurally controlled by the presence of conduits in karstified limestones of the Waulsortian Limestone Fm. These conduits allow for the development of vertical or subvertical flow, with little lateral dissipation, which can facilitate the rapid transport of recharge fluids to depth, or facilitate the rapid ascent of thermal fluids to the surface. Evidence of these structures beneath the spring has been provided by the 3D resistivity model. Given the proven efficiency of the Cenozoic strike-slip faults at transmitting large quantities of thermal groundwater, it is likely that this hydrothermal circulation pattern is operating along the plane of the N–S structure indicated in Fig. 8, although this model fails to resolve this structure at depths in excess of 400 m.

The geothermal gradient of Ireland is poorly understood but improving; however, an average near-surface value of 25–30 °C/km is suggested for Ireland—Goodman et al. (2004); Blake et al. (2020). In the summer (system I in Fig. 7), the intermediate-temperature thermal waters come from a confined or semiconfined limestone aquifer and have a temperature that is approx. 6 °C above average. Using a simple calculation, and assuming no mixing and no loss of heat as the fluids ascend to the surface, the confined source aquifer for this flow system is likely to be situated at a minimum depth of 240 m. System II in Fig. 7 represents the mixing of these intermediate-temperature waters with cool, fresh, recharge waters as the regional water table rises and activates shallow, cold, flow systems with the onset of winter. In the winter, the high-temperature thermal waters of system III are approx. 12 °C above average, which represents a minimum depth of circulation of 500 m (again assuming no mixing and no loss of heat). The rapid onset of the high-temperature winter thermal system is probably caused by piston flow in karst conduits as the winter hydrothermal circulation pattern is activated by a large influx of recharge waters. The hydrochemistry of these waters suggests that all flow systems in Fig. 7 operate in limestone bedrock. The resistivity model suggests a thickness of limestone of approximately 1,000 m; therefore, a deep hydrothermal circulation pattern within limestone to depths in excess of 500 m is feasible.

Deep groundwater circulation in limestone must occur along dissolutionally enhanced fractures and faults, as primary porosity is usually very low. The number of hydraulically transmissive fractures is expected to decrease with depth and so the transmission of water will occur in highly localised zones within the rock. Fluids will be more likely to flow along open fractures (i.e., those that are opened by tectonic forces). It is therefore possible to speculate that regional, deep, groundwater circulation in the Dublin Basin will occur in karstified zones, along vertically pervasive NNW Cenozoic strike-slipfaults and older Carboniferous normal faults. Gunn et al. (2006) carried out isotopic studies on deep groundwater circulation in a carbonate aquifer in Derbyshire, England, and calculated that deep, thermal, groundwater flow constitutes 5% of total groundwater discharge from the Peak District limestone aquifer. A similar study for the wider Dublin Basin would be a valuable addition to understanding regional thermal groundwater flow, and perhaps enable quantification of thermal flow in the region.

## Conclusions

The results of a multidisciplinary investigation of the unusually variable St. Gorman’s Well thermal spring has allowed for the development of a conceptual model for the operation of this complex hydrogeological feature. Three distinct patterns of behaviour have been observed from detailed temperature and EC profiles. These patterns have annual repeatability and represent the interaction of three different flow systems within the overall hydrothermal circulation pattern. They indicate a nonlinear hydrogeological system with rapid changes from one pattern of behaviour to another, and little direct influence from rainfall. This suggests a degree of storage in the flow system, and insulation from shallow recharge processes during the summer when the regional water table is lower. The semidiurnal signature in temperature, EC and water level indicates confined conditions in the thermal aquifer for at least part of the year. The results of a previous hydrochemical analysis (Blake et al. 2016a) suggest that the thermal waters flow entirely in limestone bedrock, and subtle variations in the minor and trace element chemistry are due to the interaction of different flow systems, as identified from the time-lapse temperature profile.

The results of a 3D inversion of AMT data from St. Gorman’s Well reveal a compelling N–S oriented region of reduced resistivity, which is interpreted as a water-bearing Cenozoic strike-slip fault. This structure, in combination with the NW–SE Carboniferous normal fault to the southwest of the spring, is likely to be the main pathway of a relatively deep hydrothermal circulatory system. The results of the 3D model are corroborated by borehole records from the area. The conceptual model positions the structurally- and recharge-controlled hydrothermal system entirely in limestone bedrock. In the summer, the intermediate temperatures are provided by a deep, confined aquifer at a minimum depth of 240 m. In winter, the high temperatures and high discharges are provided by the seasonal activation of a deep hydrothermal circulation pattern in the limestone, at a minimum depth of 500 m. This circulation pattern may extend within the limestone to depths of approximately 1,000 m.

It is evident that the development of karst along intersecting structures within the Waulsortian Limestone Fm. has been the main factor in the development of a thermal spring at St. Gorman’s Well. It is likely that the particular properties of these limestones, when they are intersected by major geological structures, have played an important role in the development of thermal springs elsewhere in Ireland. If the thermal springs are to be exploited in the future for geothermal energy purposes, it is vital to gain a thorough understanding of the structural geology of the spring sites, at shallow and deep levels, in order to effectively target this geothermal resource. This study documents how a geophysical technique such as AMT can assist in this regard. Since the hydrothermal circulation patterns are likely to be centred on the intersection of geological structures, a 3D deployment of stations followed by 3D inversion could be an optimal strategy for future AMT surveys at similar sites.

## Supplementary Information

ESM 1 (PDF 1360 kb)
