# Supplementary material for: Characterising thermal water circulation in fractured bedrock using a multidisciplinary approach: a case study of St. Gorman’s Well, Ireland
Source: Hydrogeol J. 2021 Oct 5;29(8):2595–611. doi: 10.1007/s10040-021-02393-1 (PMC8613175; doi:10.1007/s10040-021-02393-1)
Supplement: Supplementary file 1 — (PDF 1360 kb) [file 10040_2021_2393_MOESM1_ESM.pdf]

Electronic supplementary material – Hydrogeology Journal

Characterising thermal water circulation in fractured bedrock using a multi-disciplinary approach: a case study of St. Gorman's Well, Ireland

Sarah Blake[1,3,4*], Tiernan Henry[2,3], John Paul Moore[3], John Murray,[2,3], Joan Campanyà[1], Mark R. Muller[1], Alan G. Jones[1], Volker Rath[1], John Walsh[3]

1. Dublin Institute for Advanced Studies (DIAS), Merrion Square, Dublin 2, Ireland.
2. Earth and Ocean Sciences, School of Natural Sciences, National University of Ireland Galway, University Road, Galway, Ireland.
3. Irish Centre for Research in Applied Geosciences (iCRAG).
4. Geological Survey Ireland, Beggars Bush, Haddington Road, Dublin 2, Ireland.

*Corresponding author email: sarah.blake@gsi.ie

S1. Methodology

S1.1. Temperature and electrical conductivity measurements

A HOBO U24-001 temperature and conductivity logger was installed in the borehole at St. Gorman's Well in July 2013 and recorded temperature (°C) and EC (µS/cm) at 15-minute intervals. The logger was calibrated before installation and cross-checked against field measurements of temperature and EC each time the data were collected. Raw, unadjusted EC measurements are presented in Fig. 8 of the main article. In general, the temperature and EC readings were reliable and seemed unaffected by the influence of fouling by bacterial growths on the sensors. Temperature and EC data are missing for the period between July 28th and August 6th 2013 due to instrument failure. Water level measurements were also recorded every 15 minutes from late April 2014 using a Solinst Levelogger LT unit, which was suspended downhole at a depth of approximately 6 m below ground level. The measurements were compensated for barometric pressure using measurements from a Solinst Barologger LT unit

positioned in a nearby barn. Water level measurements are presented in metres above an arbitrary datum; in this case the datum is the position of the logger downhole.

S1.1.1 Hydrochemical measurements and analysis

In the course of the IRETHERM project, samples were collected and analysed from the six thermal springs shown in Fig. 1c. These hydrochemical analyses are the subject of a detailed study in Blake et al. (2016). Geographical coordinates, geological setting, maximum temperatures and a brief description of each spring is provided here in Table S1. Data were recovered for analysis over five seasons to assess the temporal variation in the spring chemistry and to provide some seasonal overlap for a more robust analysis. The springs were sampled in July/August and October 2013, and in January, May and August 2014 (see Fig. 6 of main article). Temperature, electrical conductivity, and pH were recorded at each spring prior to sampling using a calibrated YSI 556 portable multi-probe and a Hanna HI 98130 Combo meter (use of both instruments facilitated cross-checking of results).

The samples were analysed for major and minor ions by ELS Ltd., Cork, Ireland, and also for a suite of 70 trace elements by Acme Ltd. (now trading as Bureau Veritas Ltd.), Vancouver, Canada. Samples were collected using a low-flow peristaltic pump and an in-line 0.45 µm filter to obtain samples that were most representative of the formation water (see Henry, 2014). The method used was based upon the technical standards ASTM D6452-99 and ISO 5667-11, and the methods of Barcelona et al. (1994) and Puls and Barcelona (1996). Samples for trace element analysis were acidified to a pH of < 2 with trace metal grade nitric acid (John, 2000; Huang et al., 2013) prior to transport. All spring samples were duplicated for each round and a system of blanks using ultrapure water (from a Milli-Q® water purification system) was analysed by each laboratory for each round (as recommended by USGS, 2006). The blanks showed no contamination of the samples. The samples were stored in a cool box during transport and storage prior to shipment to the laboratories for analysis.

At ELS Ltd. the analyses for total concentrations of major ions were measured using inductively coupled plasma mass spectrometry (EM130 ICP-MS), total alkalinity was measured using a Titralab

EW153, and sulfate and chloride concentrations were determined using EW154M-1 AQ2-UP2 EW015/016 Autoanalyser Spectrophotometry. At Acme Ltd. trace element concentrations were determined using ICP-MS. Details of the analytes, including limits of quantification (LOQ), are presented in Table S2.

As a check on the accuracy of the analytical results, ionic balance errors were calculated using PhreeqC (version 2.18) (Parkhurst and Appelo, 1999) with the minteq.dat database. The majority of samples had calculated errors below the recommended standard of ±5% (Freeze and Cherry, 1979), with 14 % of the samples having elevated errors of between ±5% and ±10%. All samples were retained for further analysis as the ionic balance error of 10% was deemed acceptable (e.g., Cloutier et al., 2008; King et al., 2014).

	Location	Geological setting	Max. T (°C)	Description
St. Edmundsbury spring	53°21'58.59"N 6°25'35.42"W	Waulsortian Limestone Formation	17	Discharges from bedrock on south bank of River Liffey. Flooded periodically (minimum temperature not representative of thermal groundwater). Conspicuous iron staining from spring waters.
St. Gorman's Well	53°26'34.57"N 6°53'9.68"W	Adjacent to faulted contact between Waulsortian and Lucan Formation limestones	21.8	Ephemeral pond, adjacent borehole used for sampling - drilled in 1980s. Normal flow pattern is artesian in winter (max. ~ 1,000 m^3/d) when pond is full. Maximum temperatures in winter.
Huntstown Fault spring	53°24'11.44"N 6°19'54.29"W	Strike-slip fault of Tertiary age in Boston Hill Fm. limestone	16.3	Discharges from 1 m wide cavity along fault. Steady temperature and maximum discharges of ~ 5,000 m^3/d reported.
Kemmins Mill spring	53°25'47.13"N 6°38'35.16"W	Gravelly till deposits overlying faulted contact between Lucan Fm. and older limestones	14.9	Shallow abstraction well in gravel deposits used for domestic and farming purposes. Steady temperature and "slow boil" bubbling.
Kilbrook spring	53°25'24.23"N 6°46'31.63"W	Gravel and sand glacial till over faulted contact between Lucan Fm. and younger Namurian deposits	25	Discharges from old gravel quarry excavations. Depth to bedrock estimated at 25 - 30 m. Discharge (max. ~ 850 m^3/d) greatest in winter. Fairly steady, high temperature throughout the year.
Louisa Bridge Spa Well	53°22'14.44"N 6°30'23.42"W	Gravel deposits overlying Lucan Fm. limestone	17.5	Historical spa well and pond with engineered surrounds built in early-19th century. Steady temperature, yellowish-orange deposits left by spring water.

Table S1: Morphological and geological setting of the Leinster thermal springs surveyed for the IRETHERM project (see Fig. 1c in the main article). Temperature data measured using the HOBO temperature loggers.

	Analyte	Measured units	LOQ	Method of analysis
**Major ions**	Ca	ppm	0.05	ICP-MS
(> 1 ppm)	Cl	ppm	1	ICP-MS
	K	ppm	0.05	ICP-MS
	Mg	ppm	0.05	ICP-MS
	Na	ppm	0.05	ICP-MS
	Sr	ppb	0.01	ICP-MS
	HCO_3	ppm $CaCO_3$	10	Titralab
	SO_4	ppm	1	Spectrophotometry
	Si	ppm	1	Spectrophotometry
**Minor & trace ions**	NH_3	ppm N	0.007	Spectrophotometry
	NH_4	ppm	0.009	Spectrophotometry
	F	ppm	0.1	Ion chromatography
	As	ppb	0.5	ICP-MS
	B	ppb	5	ICP-MS
	Ba	ppb	0.05	ICP-MS
	Br	ppb	5	ICP-MS
	Co	ppb	0.02	ICP-MS
	Cs	ppb	0.01	ICP-MS
	Cu	ppb	0.1	ICP-MS
	Li	ppb	0.1	ICP-MS
	Mn	ppb	0.05	ICP-MS
	Mo	ppb	0.1	ICP-MS
	P	ppb	10	ICP-MS
	Rb	ppb	0.01	ICP-MS
	Rh	ppb	0.01	ICP-MS
	Sb	ppb	0.05	ICP-MS
	Se	ppb	0.5	ICP-MS
	Tl	ppb	0.01	ICP-MS
	U	ppb	0.02	ICP-MS
	Zn	ppb	0.5	ICP-MS

Table S2 (previous page): Details of the hydrochemical analysis; major, minor and trace analytes, limits of quantification and methods of analysis (from Blake et al., 2016).

**Water balance for St. Gorman's Well**	
	**WL Fm.**
Recharge (mm/yr)	277
Eff. Rainfall (mm/yr)	447
Max recharge capacity	200
Max discharge of well (m^3/d)	1100
Radius of influence (m) water	6794.21
Area of influence (m^2) water	144946633
Effective porosity	0.01
Area of influence (km^2)	**144.95**

Table S3: Simplistic water balance calculation for maximum discharge at St. Gorman's Well. Assuming all of the groundwater comes from a karst limestone aquifer in the Waulsortian Limestone Fm., and an effective porosity of 0.01 (based upon lower end of range of values for regional karstic aquifers), the thermal spring has a contributory area of 145 km^2, which could feasibly be contained within the Blackwater (Longwood) sub-catchment (area of 181 km^2).

S2. Audio-magnetotelluric survey

S2.1 Dimensionality analysis of AMT data

The dimensionality of the data was analysed by investigating the **Z** and **T** responses independently of each other. For the **Z** responses, the dimensionality analysis was performed by examining the phase tensors (Caldwell et al., 2004), which have the advantage of being unaffected by galvanic distortion of the electric fields. Figure S1 shows the calculated phase tensor for each frequency for each station, depicted as an ellipse. For a 1-D scenario the phase tensor will be represented by a circle, and for a 2-D case the phase tensor will be represented by a symmetrical ellipse, with the orientation of the major axis aligned either parallel or perpendicular to the regional geoelectrical strike direction. For 3-D cases the phase tensor will be non-symmetrical, necessitating the use of an additional angle, β, to

characterise the tensor. In Figure S1, the ellipses representing 3-D conditions are coloured depending upon the magnitude of β normalized by the corresponding error, following the approach of Campanyà et al. (2016). All stations in Figure S1 show coloured ellipses for some frequencies, indicating 3-D conditions for the survey area. For the **T** responses, induction arrows (Schmucker, 1970) following the Parkinson criteria (i.e., the real arrows tend to point towards current concentrations in conductive anomalies (Jones, 1986)) were used. Figure S2 shows the induction arrows for each station and each frequency (station 33 has no induction arrows because the **T** data quality was poor for this station). For a 1-D scenario the length of the induction arrows will be less than the threshold length of the assumed errors as there is no induced vertical magnetic field. For a 2-D scenario the induction arrows will point in the same or exactly opposite directions for all periods and stations. In a 3-D scenario, real and imaginary induction arrows will point in different (oblique) directions at any one frequency for any station (as can be seen in Figure S2). The results from Figures S1 and S2 indicate the existence of a 3-D scenario beneath the survey area.

S2.2 3-D inversion of AMT data

AMT data from 28 frequencies (excluding frequencies in the dead-band, particularly between 800 Hz and 2,000 Hz) were prepared for the inversion; these data were subsequently re-edited on a station-by-station basis to remove particularly noisy frequencies. The data were inverted using the ModEM 3-D inversion code (Egbert and Kelbert, 2012; Kelbert et al., 2014). The vertical magnetic transfer functions (**T**) were inverted alongside the four components of the impedance tensors (**Z**) to improve the resolution of the subsurface resistivity values (e.g., Siripunvaraporn and Egbert, 2009). The mesh for the resistivity model consisted of 90 × 90 × 90 cells, with square cells with sides 50 m long in the horizontal plane of the central region of interest. This central region was a square with sides 3 km long. Padding cells were added in the x and y directions with an incremental factor of 1.3. In the z direction, 10 air layers were added above the resistivity model. The first (surface) layer of the model was 10 m thick; these layers were incrementally increased by a factor of 1.025 until a thickness of 60 m was achieved. The layers were then increased by a factor of 1.1. The final model dimensions were 8 km × 8 km × 5 km. Several preliminary models were assigned a homogeneous half space with

varying resistivity values as their starting and prior models; the best results (i.e., with the least extreme values and resolving the most structure) were obtained with half-spaces of 300 and 500 Ωm (0.003 and 0.002 S/m). An average of four models (two starting models with homogeneous half-spaces of 300 Ωm and 500 Ωm, and the two resultant models from those inversions) was calculated and set as the prior model for the final inversion. The model mesh was not rotated as preliminary models showed the subsurface to have 3-D structure with no one predominant geoelectrical strike direction evident. An error floor of 5 % was imposed for all components of **Z** (calculated from the modulus of the off-diagonal components $\mathbf{Z}_{xy}$ and $\mathbf{Z}_{yx}$), and an absolute error of 0.03 was used for **T**. Variation of the smoothing parameters was investigated for the model; values between 0.1 and 0.5 were tested, and an intermediate value of 0.3 (in all directions) for the smoothing parameter gave the minimum normalised root mean square (nRMS) misfit for the data.

No correction or compensation was applied to the data to account for galvanic distortion, which is a tractable problem in 2-D cases, but far less practicable in 3-D (see Jones, 2011). An examination of the apparent resistivity curves revealed no particular "problem areas" for galvanic distortion. As a 3-D modelling approach was used, with a fine parameterization in the uppermost part of the model, it was expected that the model would not be greatly affected by near-surface galvanic distortion effects at our target depths (e.g., Sasaki and Meju, 2006; Farquharson and Craven, 2009; Meqbel et al., 2014). Galvanic distortion may affect the very shallowest layers of the model, but at depth, particularly beneath 100 m where every part of the model is sampled by numerous stations, the conductivity shows a smooth and consistent distribution and any unresolved features near to the surface appear to have been assimilated by the model. Hence, at depths greater than 100 m the effects of galvanic distortion in the model should be negligible. Also, the inversion of **T** alongside **Z** should decrease the susceptibility of the model to the effects of galvanic distortion. As **T** does not involve the electric field directly, it is not subject to the same galvanic distortion as **Z**. However, it can become distorted if the deflection of electric currents by in-phase electric fields alters the vertical magnetic fields (Booker, 2014). The resulting models do not show obvious artefacts (i.e., site-correlated model structures), which commonly indicate the presence of static shifts.

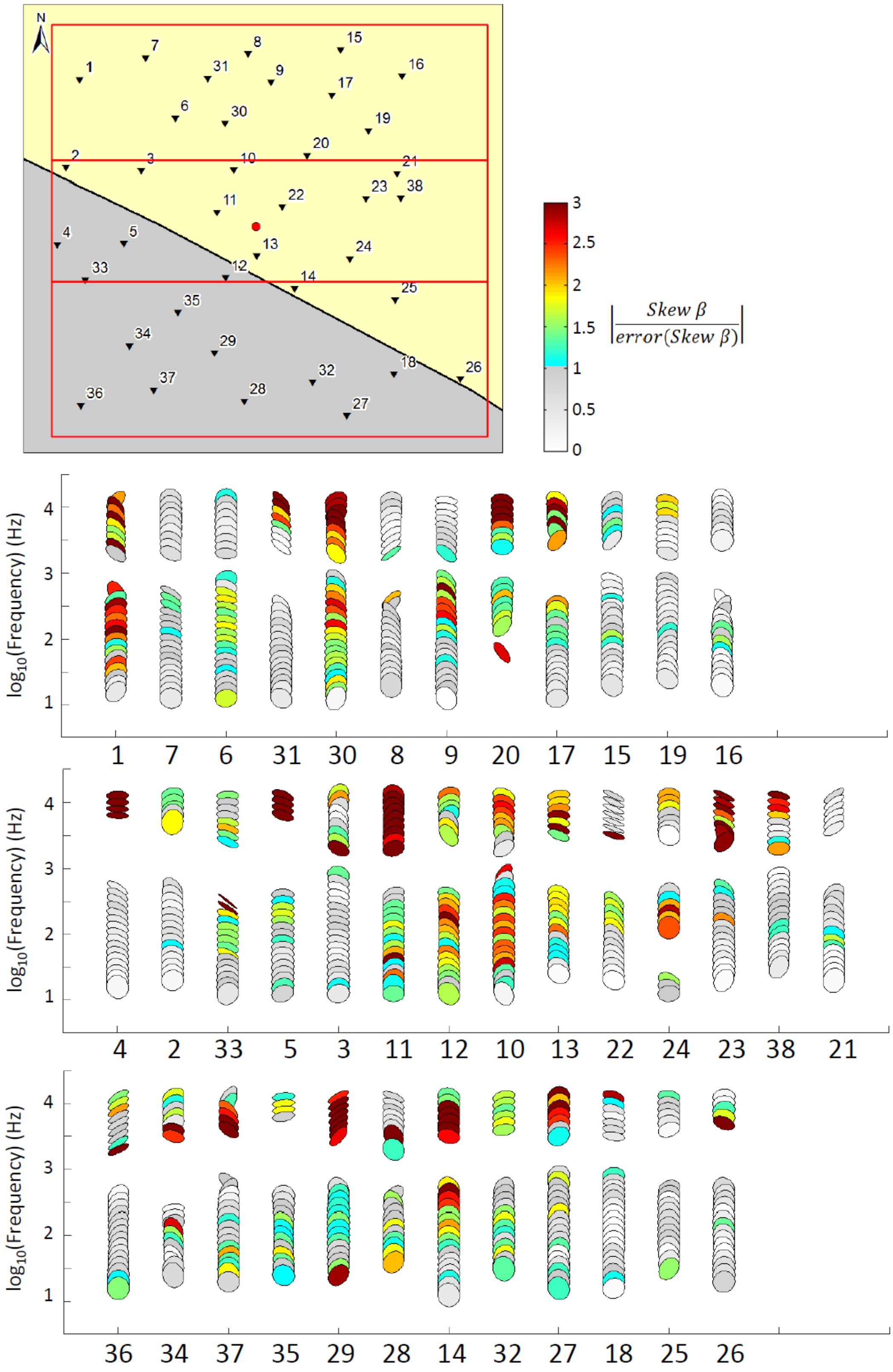

Figure S1: Phase tensor dimensionality analysis using Z responses. White-grey colours indicate frequencies affected by the presence of 1-D or 2-D structures. Other colours represent frequencies affected by 3-D structures. Stations are arranged from W to E in three panels to correspond with the boxes outlined in the map of the survey area (see Fig. 2 in main article).

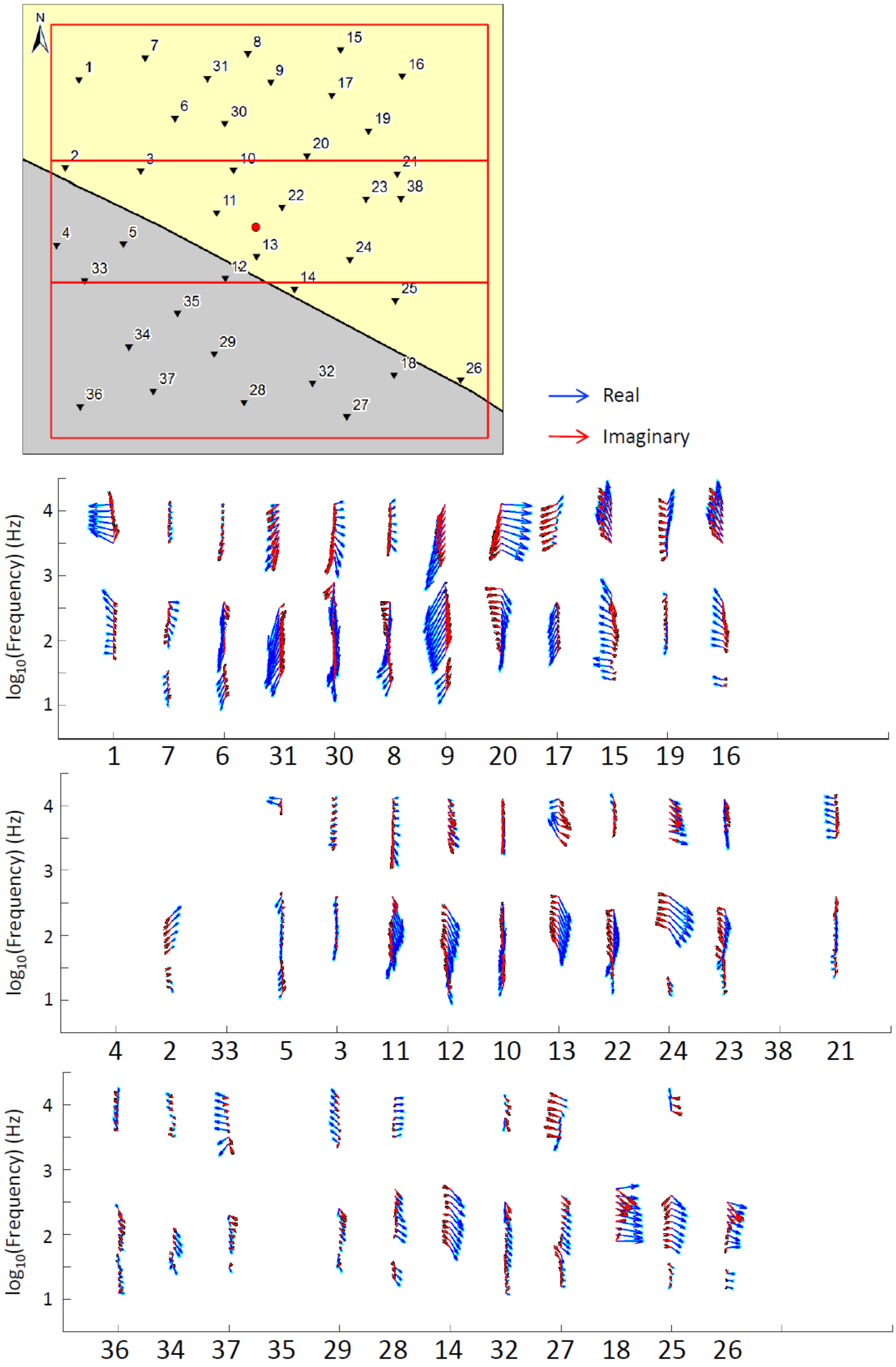

Figure S2: Induction arrow dimensionality analysis using **T** responses, following the Parkinson criteria. Stations are arranged from W to E in three panels to correspond with the boxes outlined in the map of the survey area (see Fig. 2 in main article).

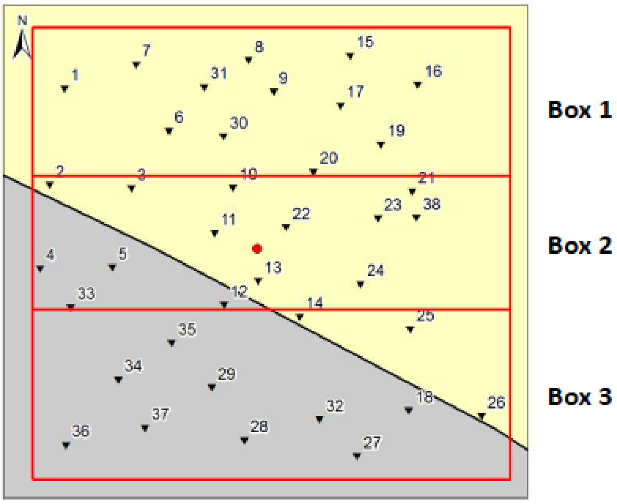

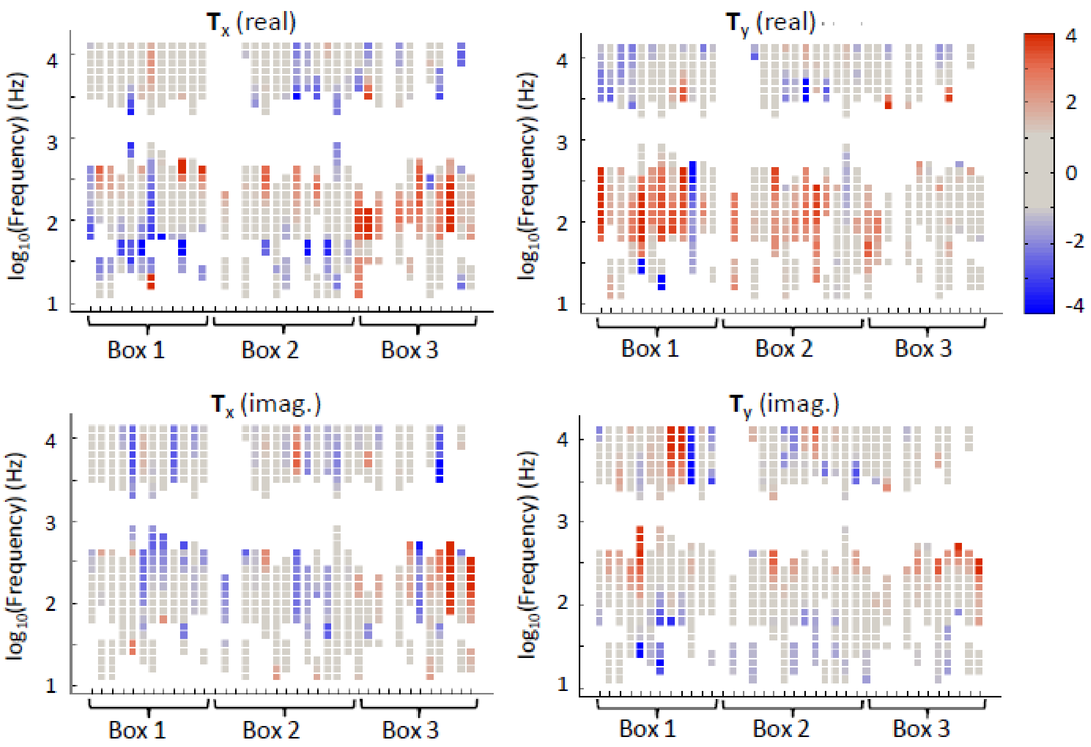

Figure S3: (cont. overleaf) Representation of the data fit to the model responses from the final 3-D inversion of AMT data. Data from both components of T and all four components of Z are represented (real and imaginary parts). The stations are grouped to reflect the three boxes in the inset: the stations are arranged in order of their appearance from W to E. The coloured scale represents the difference between the data and the model response divided by the error for each frequency at each station.

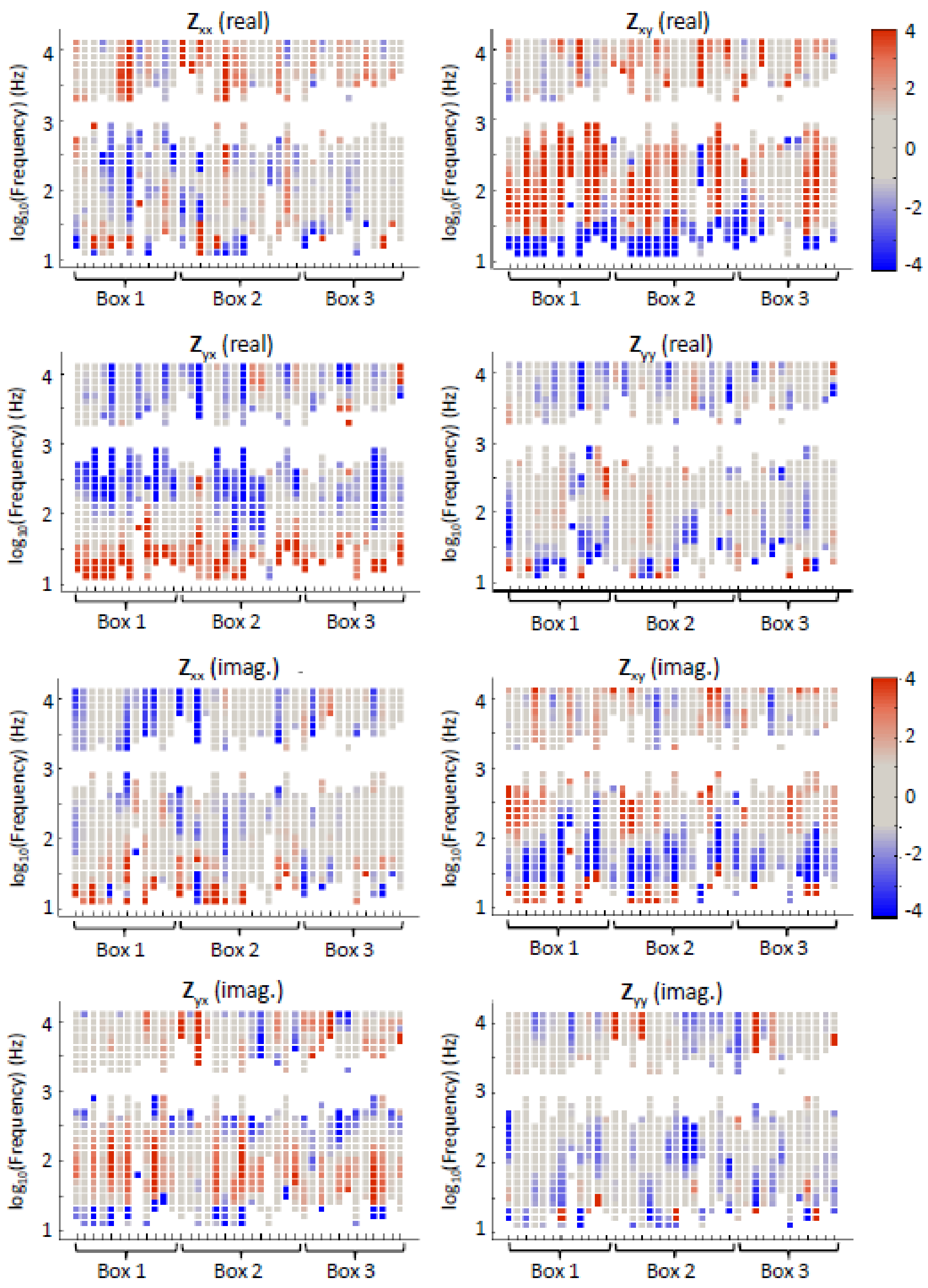

Z_{xx} (real)
Z_{xy} (real)
Z_{yx} (real)
Z_{yy} (real)
Z_{xx} (imag.)
Z_{xy} (imag.)
Z_{yx} (imag.)
Z_{yy} (imag.)
$\log_{10}$(Frequency) (Hz)
Box 1
Box 2
Box 3
4
2
0
-2
-4

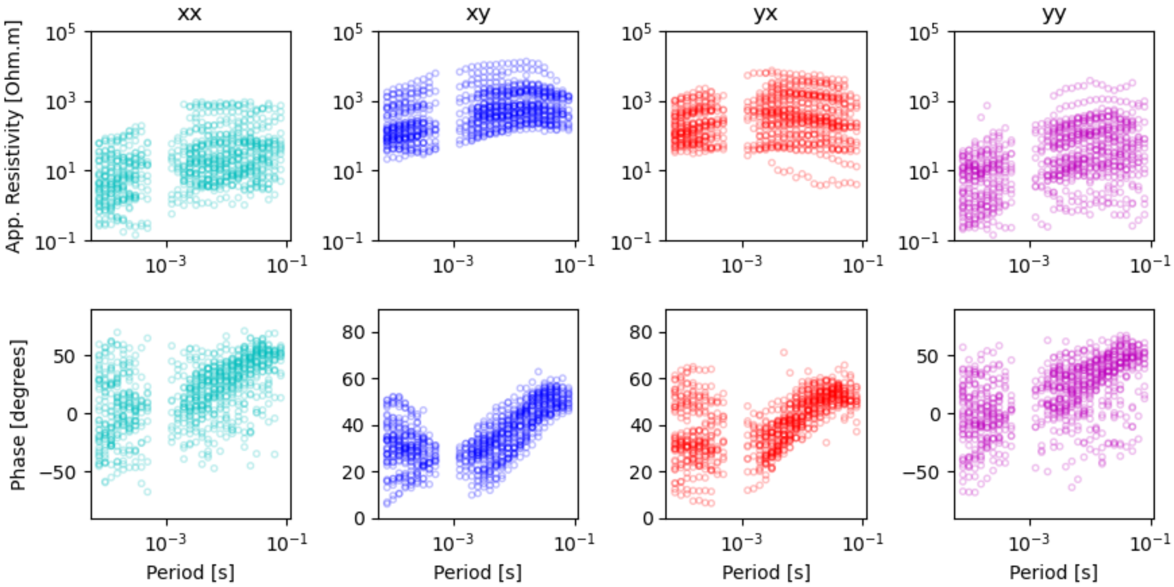

Figure S4: Plot of phase and apparent resistivity against period for all stations.

ESM References

ASTM International, 2005. Standard Guide for Purging Methods for Wells Used for Groundwater Quality Investigations (D6452-99). ASTM International, West Conshohocken, Pennsylvania.

Barcelona, M.J., Wehrmann, H.A., Varljen, M.D., 1994. Reproducible well purging procedures and VOC stabilization criteria for ground water sampling. Ground Water 32 (1), 12-22.

Blake, S., Henry, T., Murray, J., Flood, R., Muller, M.R., Jones, A.G, Rath, V., 2016. Compositional multivariate statistical analysis of thermal groundwater provenance: A hydrogeochemical case study from Ireland. Applied Geochemistry 75, 171-188.

Booker, J.R., 2014. The magnetotelluric phase tensor: a critical review. Surveys in Geophysics 35, 7-40.

Caldwell, G.T., Bibby, H.M., Brown, C., 2004. The magnetotelluric phase tensor. Geophysical Journal International 158, 457-469.

Campanyà, J., Ogaya, X., Jones, A.G., Rath, V., Vozar, J., 2016. The advantages of complementing

MT profiles in 3-D environments with geomagnetic transfer function and inter-station horizontal magnetic transfer function data: results from a synthetic case study. Geophysical Journal International 207 (3), 1818-1836.

Cloutier, V., Lefebvre, R., Therrien, R., Savard, M.M., 2008. Multivariate statistical analysis of geochemical data as indicative of the hydrogeochemical evolution of groundwater in a sedimentary rock aquifer system. Journal of Hydrology 353, 294-313.

Egbert, G.D., Kelbert, A., 2012.Computational recipes for electromagnetic inverse problems. Geophysical Journal International 189, 251-267.

Farquharson, C.G., Craven, J.A., 2009. Three-dimensional inversion of magnetotelluric data for mineral exploration: an example from the McArthur River uranium deposit, Saskatchewan, Canada. Journal of Applied Geophysics 68, 450-458.

Freeze, R.A., Cherry, J.A., 1979. Groundwater. Prentice Hall, New Jersey.

Henry, T., 2014. An Integrated Approach to Characterising the Hydrogeology of the Tynagh Mine Catchment, County Galway, Ireland. Ph.D. thesis, National University of Ireland Galway.

Huang, G., Sun, J., Zhang, Y., Chen, Z., Liu, F., 2013.Impact of anthropogenic and natural processes on the evolution of groundwater chemistry in a rapidly urbanized coastal area, South China. Science of the Total Environment 463-464, 209-221.

ISO, 2006. ISO 5667-1: Water Quality – Sampling – Part 2: Guidance on Sampling Techniques. NSAI Dublin, Ireland.

John, F.B., 2000. Collecting Water-Quality Samples for Dissolved Metals-In-Water. U.S. Environmental Protection Agency, Region 6.

Jones, A.G.,1986. Parkinson's pointers' potential perfidy! Geophysical Journal of the Royal Astronomical Society 87, 1215-1224.

Jones, A.G., 2011. Three-dimensional galvanic distortion of three-dimensional regional conductivity structures: Comment on "Three-dimensional joint inversion for magnetotelluric resistivity and

static shift distributions in complex media" by Yutaka Sasaki and Max A. Meju, Journal of Geophysical Research-Solid Earth, 116.

Kelbert, A., Meqbel, N., Egbert, G.D., Tandon, K., 2014. ModEM: A modular system for inversion of electromagnetic geophysical data. Computers and Geosciences, 66, 40-53.

King, A.C., Raiber, M., Cox, M.E., 2014. Multivariate statistical analysis of hydrochemical data to assess alluvial aquifer-stream connectivity during drought and flood: Cressbrook Creek, southeast Queensland, Australia. Hydrogeology Journal 22, 481-500.

Meqbel, N.M., Egbert, G.D., Wannamaker, P.E., Kelbert, A., 2014. Deep electrical resistivity structure of the northwestern U.S. derived from 3-D inversion of US Array magnetotelluric data. Earth and Planetary Science Letters 402, 290-304.

Parkhurst, D.L., Appelo, C.A.J., 1999. User's guide to PHREEQC (version 2) – a computer program for speciation, batch-reaction, one-dimensional transport, and inverse geochemical calculations. U.S. Geological Survey Water-Resources Investigations Report 99-4259.

Puls, R.W., Barcelona, M.J., 1996. Low Flow (Minimal Drawdown) Ground-Water Sampling Procedures. US Environmental Protection Agency, Washington D.C. Report No. 540/S95/504.

Sasaki, Y., Meju, M.A., 2006. Three-dimensional joint inversion for magnetotelluric resistivity and static shift distributions in complex media. Journal of Geophysical Research – Solid Earth 111, 11 pp.

Schmucker, U., 1970. Anomalies of geomagnetic variations in the Southwestern United States.Journal of Geomagnetic Oceanography, University of California Press, Berkeley, USA.

Siripunvaraporn, W., Egbert, G., 2009. WSINV3DMT: vertical magnetic field transfer function inversion and parallel implementation. Physics of the Earth and Planetary Interiors, 173, 317-329.

USGS, 2006. National Field Manual for the Collection of Water-Quality Data: Chapter A4 Collection of Water Samples (Version 2.0).US Geological Survey TWRI Book 9. USGS, Reston, Virginia.
